# A pH-Responsive Polysaccharide Hydrogel Modulates Oxygen Delivery and Immunometabolic Remodeling for Diabetic Bone Regeneration

**DOI:** 10.34133/research.1428

**Published:** 2026-10-05

**Authors:** Haifu Sun, Nanning Lv, He Dong, Wei Geng, Jing Shi, Xinhui Wu, Yonggang Li, Yu Chen, Haowen Lu, Jun Dai, Longpo Zheng, Yusen Qiao

**Affiliations:** ^1^ Department of Orthopedics, The First Affiliated Hospital of Soochow University, Suzhou 215006, Jiangsu, China.; ^2^ Department of Orthopedics, Shanghai Tenth People’s Hospital, School of Medicine, Tongji University, Shanghai 200072, China.; ^3^ Department of Orthopaedics, Lianyungang Clinical College, Xuzhou Medical University, Lianyungang Second People’s Hospital affiliated to Kangda College of Nanjing Medical University, Lianyungang 222003, China.; ^4^ Department of Orthopaedic Oncology, Shanghai General Hospital, Shanghai Jiao Tong University School of Medicine, Shanghai 200000, China.; ^5^ Department of Orthopedics, The Second Affiliated Hospital of Soochow University, Suzhou 215000, Jiangsu, China.

## Abstract

Diabetic bone defects are difficult to repair because chronic hypoxia, oxidative stress, persistent inflammation, and impaired osteoangiogenic coupling disrupt the healing cascade. Here, we report a pH-responsive polysaccharide hydrogel, GOZ6, for spatiotemporally coordinated diabetic bone regeneration. GOZ6 is formed by dynamic Schiff base crosslinking between gelatin and oxidized konjac glucomannan and co-delivers ZIF-8@CaO_2_ and 6-gingerol, integrating gated oxygen release, calcium ion delivery, antioxidative protection, and immunomodulation. The hydrogel exhibited rapid gelation within approximately 67 s, a porous architecture with a porosity of 75.78%, pH-responsive degradation, and sustained oxygen release for up to 14 d under physiological conditions. Under mildly acidic conditions, approximately 60% of 6-gingerol was released within the first 3 d, supporting early anti-inflammatory and antioxidative intervention. In vitro, GOZ6 maintained cell viability above 90%, promoted macrophage polarization toward a reparative phenotype, suppressed pro-inflammatory cytokine production, and restored oxidative metabolism. Transcriptomic and protein analyses find that JAK1–STAT3 and PI3K–AKT may serve as central pathways involved in these effects. Moreover, the GOZ6-conditioned immune microenvironment enhanced endothelial migration and tube formation, alleviated oxidative stress in bone marrow mesenchymal stem cells, restored mitochondrial function, and promoted osteogenic differentiation. In a diabetic rat calvarial defect model, GOZ6 markedly enhanced vascularized bone regeneration, collagen deposition, and osteogenic marker expression while reducing local inflammation, without evident systemic toxicity. GOZ6 therefore provides a multifunctional strategy for converting diabetic bone defects into a repair-permissive microenvironment.

## Introduction

The escalating global diabetes pandemic has precipitated a skeletal health crisis, transforming bone fragility and repair impairment from comorbidities into direct threats to healthcare sustainability. The number of people with diabetes continues to rise [1,2]. People with type 2 diabetes face a risk of fracture nearly 3 times higher than those without diabetes [3,4]. In addition, diabetes increases the risk of delayed healing and nonunion of fractures [5–7]. The Global Burden of Disease Study highlights diabetes-related complications as a major and escalating driver of global healthcare expenditure [8]. This positions diabetic bone defects as a quintessential regenerative failure disease, characterized by intersecting pathologies: chronic hypoxia, oxidative stress overload, immunometabolic dysfunction, and osteogenic signaling collapse.

The pH of the diabetic bone injury niche is not static but dynamically changes during repair. During the early inflammatory phase, hypoxia, enhanced glycolysis, lactate accumulation, inflammatory cell infiltration, and possible infection can shift the local microenvironment toward a weakly acidic state, which is commonly simulated at approximately pH 6.5 in bone injury and inflammatory biomaterial studies [9–11]. As inflammation resolves and vascular perfusion improves, the local microenvironment gradually tends to return toward physiological pH. This acidic-to-physiological pH transition provides an important pathological basis for designing GOZ6 as a pH-responsive injectable hydrogel [12].

Although current regenerative biomaterials have proven effective in the treatment of conventional bone defects [13], they have repeatedly encountered setbacks when treating complex pathological bone defects, such as those associated with diabetes [14]. Disruption of the bone regenerative microenvironment is the core pathological mechanism [15]. Bone regeneration is not an isolated event but a dynamic process involving the combined regulation of multiple cells [16,17]. Diabetic bone defects cannot be repaired simply by providing mechanical support or osteogenic factors [18]. The root cause of their difficulty in healing lies in multiple functional dysregulations: hypoxia and oxidative stress, immune and metabolic dysfunction, and a disconnect between osteogenesis and angiogenesis [9,19,20]. Macrophages play a critical role in regulating bone homeostasis [21]. In diabetes, persistent hyperglycemia, advanced glycation end products (AGEs) [22], and an oxidative stress environment cause macrophages to rely on glycolytic metabolism, maintaining pro-inflammatory M1 polarization, releasing inflammatory mediators, and leading to widespread disruption of innate immune sensing and cytokine signaling [23,24]. This immune imbalance exacerbates local oxidative stress, delays the transition to a reparative M2 macrophage state, and thereby hinders the initiation of bone repair [25,26]. Concurrently, within this adverse microenvironment, both the matrix and endothelial cells remain in a state of energy limitation and functional suppression, leading to failed bone–vascular coupling and further compromising oxygen [27] and nutrient supply to the defect site [28]. It is precisely this comprehensive dysregulation involving immune, metabolic, vascular, and osteogenic processes that constitutes the fundamental cause of the treatment-resistant nature of diabetic bone defects.

Traditional clinical and biomaterial interventions for treating diabetic bone defects are too limited in scope to effectively address the complex, dynamic pathophysiological environment associated with diabetes [29]. Standard treatments, such as internal fixation and bone grafting [30], provide some physical support but often fail due to persistent inflammation, oxidative stress, and inadequate perfusion, leading to delayed or non-union [31,32]. Bioactive strategies, such as bone-inducing growth factors, ion-releasing scaffolds, or cell therapies, may enhance osteogenic potential [33]. However, their efficacy is often diminished when macrophage-driven inflammatory processes remain unresolved and blood supply cannot be promptly restored [34]. Conversely, immunomodulatory or antioxidant approaches can temporarily alleviate the inflammatory burden but rarely restore oxygen supply or re-establish the link between angiogenesis and osteogenesis, resulting in incomplete or transient repair [35,36]. Even oxygen-generating materials, while capable of alleviating hypoxia, may exacerbate oxidative damage if hydrogen peroxide decomposition becomes uncontrolled, leading to a surge in cytotoxic H₂O₂ and local pH disturbances. Excessive oxidative stress affects bone marrow mesenchymal stem cell (BMSC) viability [37]. Taken together, these limitations indicate that bone regeneration in diabetes requires sequential, multi-axial control rather than isolated interventions [38,39].

To address the multifactorial microenvironmental barriers in diabetic bone defects, including hypoxia, persistent inflammation, oxidative stress, and impaired osteogenesis, we developed a pH-responsive polysaccharide hydrogel system, GOZ6, to achieve spatiotemporally combined regulation of the regenerative niche. This system is based on dynamic Schiff base crosslinking between gelatin and oxidized konjac glucomannan, forming an injectable hydrogel matrix with self-healing capability and pH-responsive release behavior [40]. Under the mildly acidic conditions that characterize the early inflammatory phase, GOZ6 preferentially releases 6-gingerol [41], thereby modulating macrophage metabolic status and polarization, attenuating inflammatory activation, and reducing oxidative stress. Meanwhile, the acidic microenvironment gradually triggers the degradation of zeolitic imidazolate framework (ZIF) particles [42], leading to the release of CaO_2_ for sustained oxygen generation to relieve local hypoxia while simultaneously delivering Ca^2+^ to provide osteoinductive cues.

Based on this design, we further propose a 4-axis, spatiotemporally coordinated framework for diabetic bone regeneration, comprising immunometabolic reprogramming, redox homeostasis restoration, oxygenation-driven angiogenesis, and ion-mediated osteogenesis. GOZ6 implements this concept through an injectable, self-healing polysaccharide hydrogel that integrates pH-gated oxygen release with Ca^2+^ delivery while co-delivering an immunomodulatory small molecule to redirect macrophage signaling and restore a repair-permissive immune landscape (Fig. 1). By coupling early relief of hypoxia and suppression of inflammation with subsequent vascular reconstruction and osteogenic consolidation, this 4-axis strategy is designed to transform diabetic bone defects from a self-reinforcing regeneration-hostile niche into a regeneration-supportive microenvironment, thereby enabling robust mineralization and functional recovery.

**Fig. 1.**
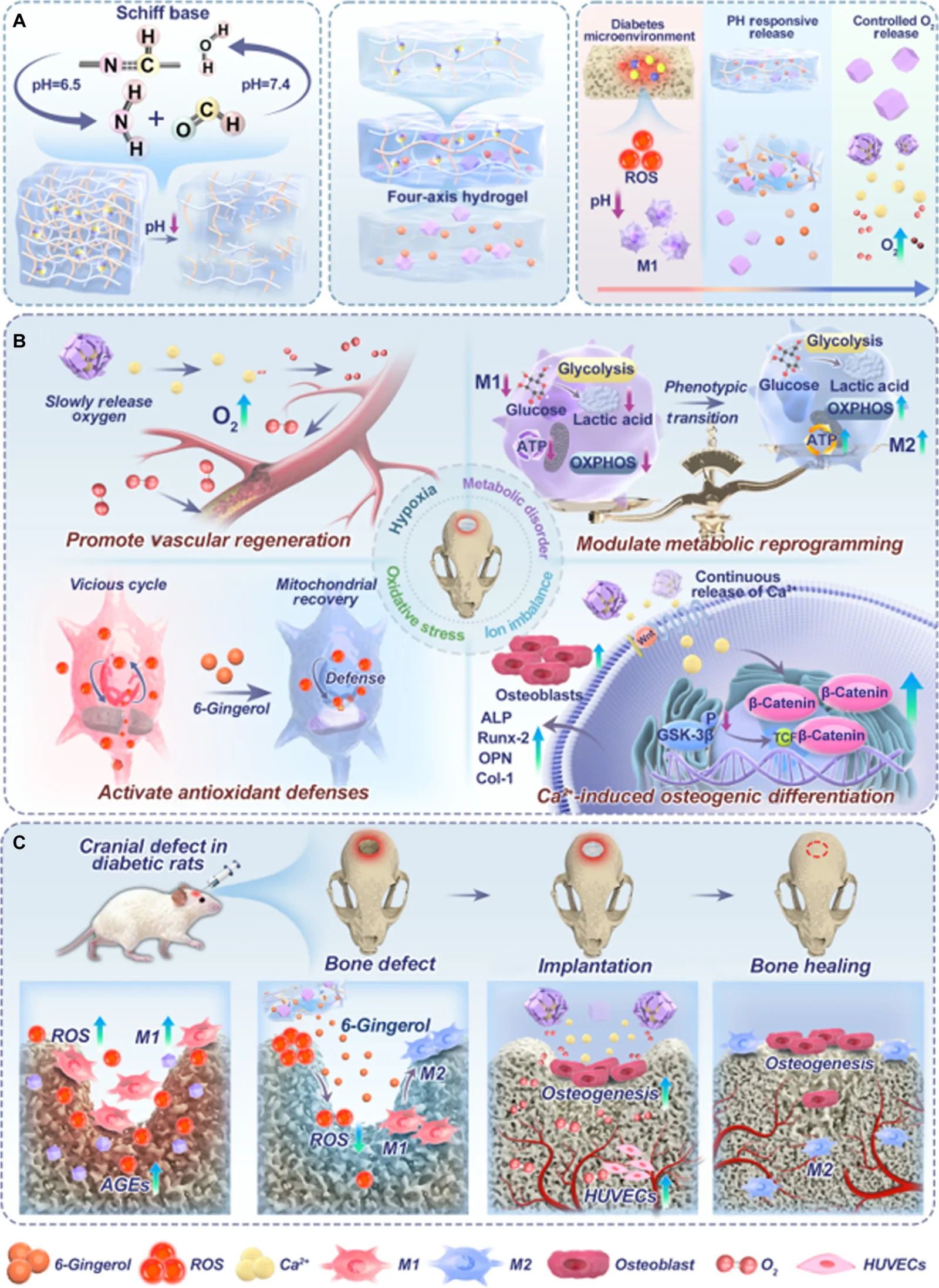
Schematic diagram of GOZ6 promoting diabetic bone regeneration. (A) GOZ6 hydrogel exhibits responsive drug release under weakly acidic conditions. (B) A 4-pronged regulatory strategy of the GOZ6 hydrogel: releasing oxygen to promote vascular regeneration, modulating macrophage polarization, reducing oxidative stress levels, and releasing ions to induce osteogenesis. (C) Schematic illustration of the GOZ6 hydrogel regulating the regeneration of cranial bone defects in diabetic rats.

## Results

### Fabrication and characterization of ZIF-8-encapsulated calcium peroxide nanoparticles

ZIF-8 is a well-established porous nanocarrier with high loading capacity. We first synthesized ZIF-8 nanoparticles and subsequently prepared ZIF-8@CaO₂ composite particles. Scanning electron microscopy (SEM) images showed that pristine CaO₂ consisted of relatively small primary nanoparticles with a comparatively uniform morphology (Fig. S1). Pristine ZIF-8 displayed a regular particulate morphology and a relatively uniform size distribution, whereas ZIF-8@CaO₂ formed larger composite particles. Energy-dispersive spectrometer (EDS) scanning shows that the ZIF-8@CaO₂ phase contains additional Ca and O atoms compared to ZIF-8 (Fig. 2A and Figs. S2 and S3). Particle size analysis showed that CaO₂ particles were small, and their size increased after the composite was formed. The diameter increased from approximately 149 nm for the original ZIF-8 to approximately 486 nm for ZIF-8@CaO₂ (Fig. 2B and Fig. S4). The increased particle size of ZIF-8@CaO₂ may result from CaO₂-induced changes in the local nucleation and growth environment of ZIF-8, together with limited association of the resulting composite particles [43]. Transmission electron microscopy (TEM) imaging showed that CaO₂ appears as nanoparticles and ZIF-8@CaO₂ contained electron-dense nanoscale regions within or closely associated with the ZIF-8 domains, supporting the incorporation of CaO₂ into the composite structure (Fig. 2C and Fig. S5). TEM-EDS mapping further detected Ca and O signals within the ZIF-8@CaO₂ particles, together with the characteristic elements of the ZIF-8 framework (Fig. 2D and Fig. S6). These morphological, particle-size, and elemental analyses collectively support the formation of ZIF-8@CaO₂ composite particles.

**Fig. 2.**
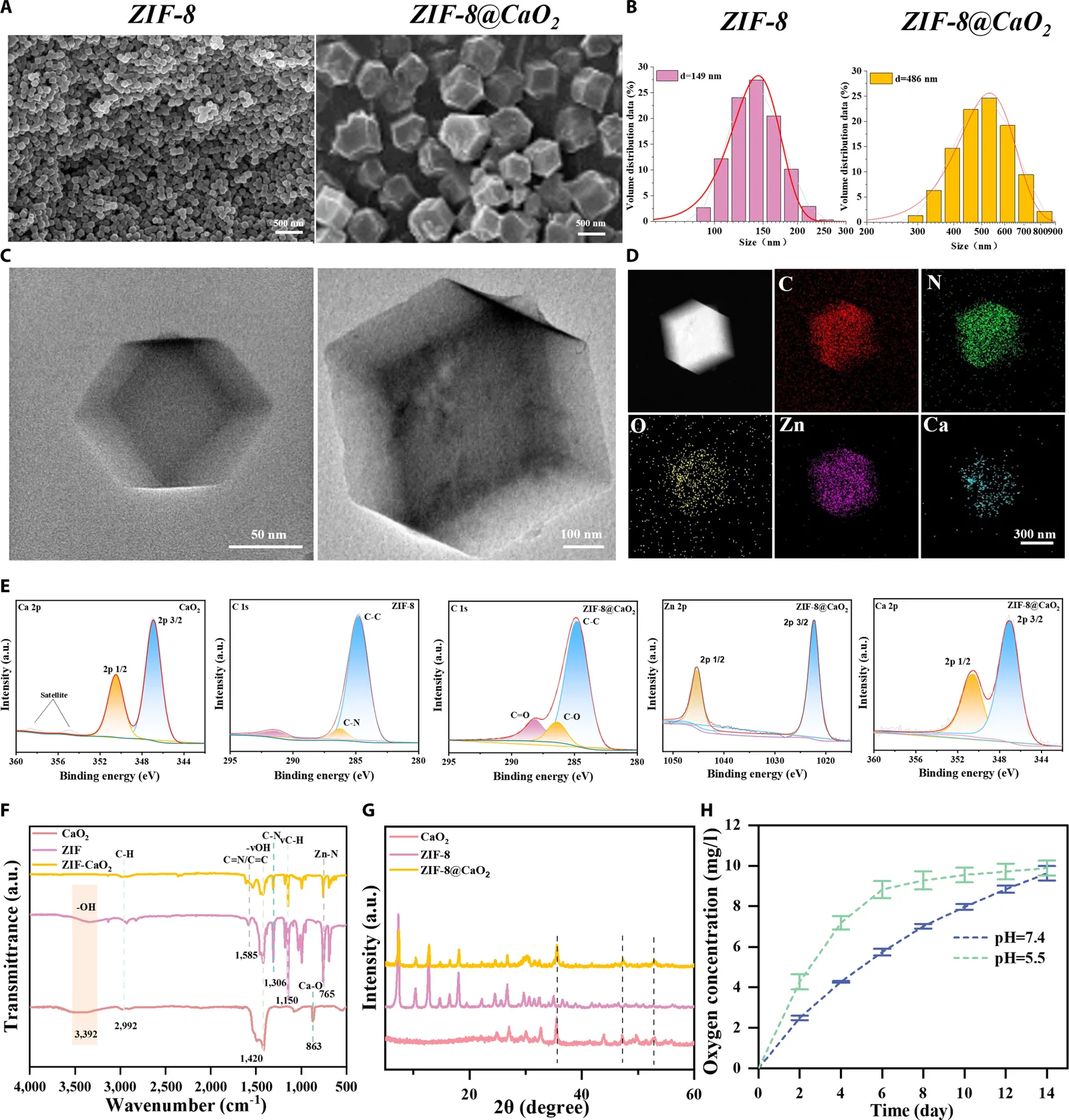
Physical and chemical characterization of ZIF-8@CaO_2_. (A) SEM images of ZIF-8 and ZIF-8@CaO_2_. (B) Particle size analysis of ZIF-8 and ZIF-8@CaO_2_. (C) TEM photographs of ZIF-8 and ZIF-8@CaO_2_. (D) EDS elemental mapping of ZIF-8@CaO_2_. (E) X-ray photoelectron spectroscopy (XPS) spectra acquired for CaO_2_, ZIF-8, and ZIF-8@CaO_2_. (F) Fourier transform infrared (FTIR) spectra acquired for CaO_2_, ZIF-8, and ZIF-8@CaO_2_. (G) XRD patterns acquired for CaO_2_, ZIF-8, and ZIF-8@CaO_2_. (H) Oxygen release curves of ZIF-8@CaO_2_ under different pH conditions. Data are presented as the mean ± SD (*n* = 3).

Successful composite formation was further verified by x-ray photoelectron spectroscopy. Compared with pristine ZIF-8, the composite group shows an additional calcium-related peak, confirming the presence of calcium-containing components in the system (Fig. 2E and Fig. S7). Fourier transform infrared (FTIR) spectroscopy provided additional structural confirmation. In the calcium peroxide group, the characteristic absorption peak at approximately 863 wavenumbers corresponds to calcium–oxygen stretching vibration, confirming the presence of calcium peroxide. In the ZIF-8 group, the peak near 1,585 wavenumbers corresponds to carbon–carbon double bond stretching vibration in the imidazole ring. The peak near 765 wavenumbers is attributed to zinc–nitrogen stretching vibration. Both peaks are characteristic of the ZIF-8 framework. Following composite formation, a new peak appears at approximately 600 wavenumbers. This peak is attributed to interactions between calcium–oxygen bonds and the ZIF-8 framework, indicating successful integration of calcium peroxide into the carrier structure (Fig. 2F). X-ray diffraction (XRD) analysis was performed to examine the crystalline structures of all 3 materials. As shown in Fig. 2G, calcium peroxide displays characteristic diffraction peaks corresponding to the 110, 112, and 103 crystal planes. ZIF-8 exhibits characteristic peaks of the 011, 002, 112, and 222 planes. Importantly, composite particles retain the characteristic diffraction peaks of both components. This indicates successful composite formation without disruption of the crystalline structures of either constituent material.

ZIF-8 degraded more rapidly at pH 6.5 than at pH 7.4. By day 7, approximately 20% of the initial mass remained under mildly acidic conditions (Fig. S8). These results indicate that a mildly acidic microenvironment accelerates ZIF-8 degradation. The oxygen release behavior of composite nanoparticles was systematically evaluated. As shown in Fig. 2H, under mildly acidic conditions, oxygen is released in a sustained rather than burst-like manner due to the protective encapsulation provided by ZIF-8. The release process is largely completed within approximately 8 d. Under physiological conditions, oxygen release is further prolonged to approximately 14 d. These results demonstrate that ZIF-8 coating effectively modulates oxygen release kinetics.

### Fabrication and physicochemical characterization of GOZ6 hydrogels

Following successful preparation of composite nanoparticles, we next constructed the hydrogel matrix. Konjac glucomannan was first oxidized to obtain oxidized konjac glucomannan. FTIR spectroscopy analysis confirmed successful oxidation of konjac glucomannan. Owing to the presence of abundant aldehyde groups, oxidized konjac glucomannan reacts with amino groups in gelatin via Schiff base chemistry, enabling rapid spontaneous gelation (Fig. 3A). Rheological analysis was performed to evaluate the gelation behavior of the hydrogel system. As shown in Fig. 3B, the hydrogel undergoes sol-to-gel transition within approximately 67 s, indicating rapid in situ gelation capability. This gelation process was visually confirmed by gross observation. The precursor solution transforms spontaneously from a flowing liquid into a stable gel state within seconds (Fig. 3C). In addition, the hydrogel exhibits moderate adhesive capacity and can adhere firmly between bone slices (Fig. S9). This suggests its potential suitability for localized defect filling and retention.

**Fig. 3.**
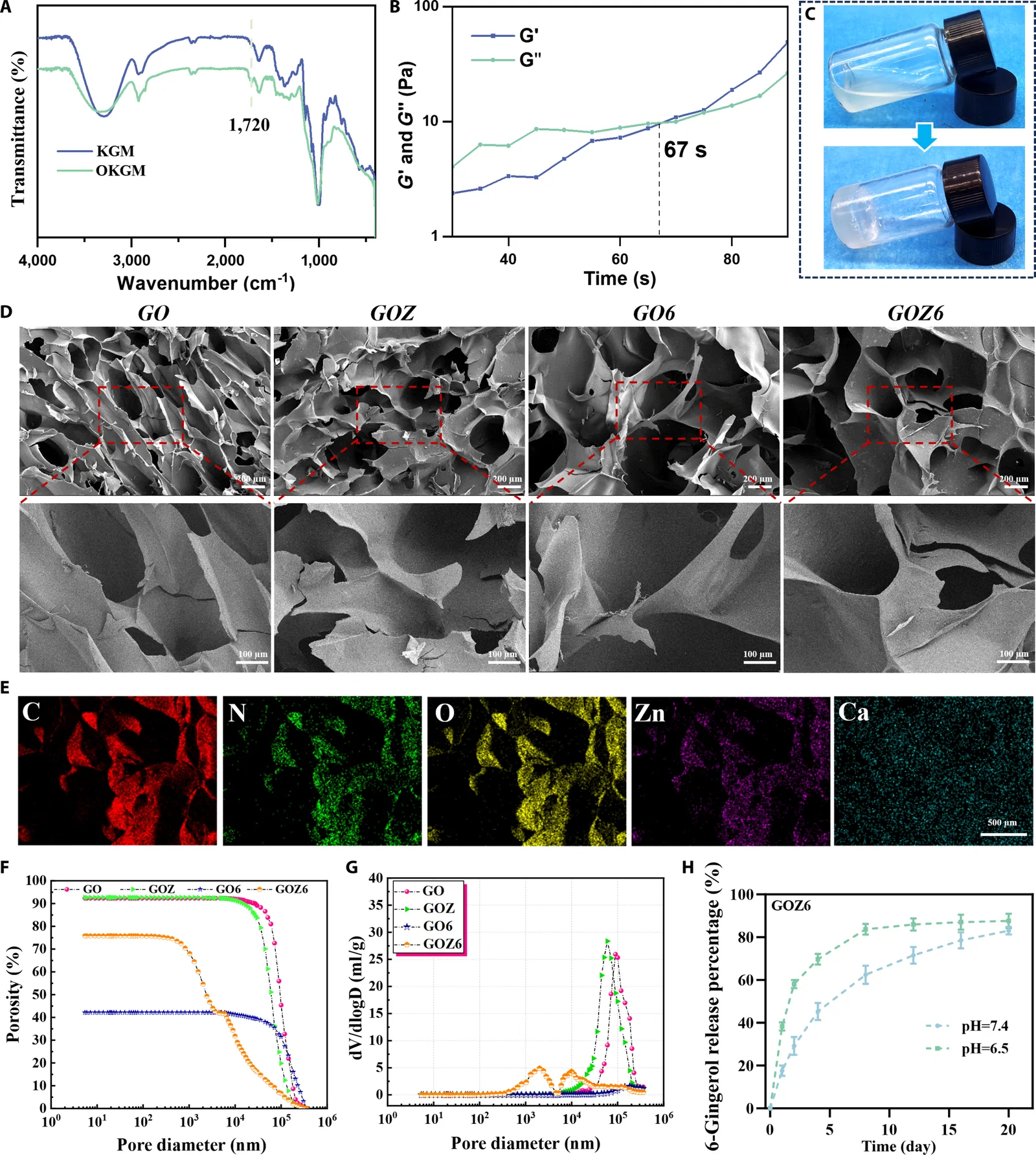
Physical characterization of GOZ6. (A) FTIR spectra acquired for the konjac glucomannan (KGM) and oxidized konjac glucomannan (OKGM). (B) Rheological testing of GOZ6. (C) Photograph of the solidified GOZ6 hydrogel. (D) SEM images of GO, GOZ, GO6, and GOZ6. (E) EDS elemental mapping of GOZ6. (F and G) Mercury intrusion porosimetry on hydrogels from each group. (H) 6-Gingerol release curves of GOZ6 under different pH conditions. Data are presented as the mean ± SD (*n* = 3).

The microstructures of the 4 hydrogel formulations were further examined by SEM (Fig. 3D). All hydrogels exhibited interconnected porous architectures with relatively uniform pore distributions. Following the incorporation of ZIF-8@CaO₂, numerous dot-like particulate structures were observed throughout the examined hydrogel regions, with no obvious large aggregates or particle-depleted areas. The addition of 6-gingerol was accompanied by an increase in mean pore size and a decrease in overall porosity. Energy-dispersive spectroscopy confirmed the expected elemental compositions of the different formulations (Fig. S10). In particular, Zn and Ca signals were broadly distributed across the GOZ6 hydrogel, consistent with the relatively uniform incorporation of ZIF-8@CaO₂ within the hydrogel network (Fig. 3E).

Mercury intrusion porosimetry was performed to further quantify pore characteristics (Fig. 3F and G). Consistent with SEM observations, the addition of 6-gingerol reduces overall hydrogel porosity. Subsequent incorporation of composite nanoparticles partially restores pore structure and increases porosity. The cumulative porosities of the GO, GO6, GOZ, and GOZ6 hydrogels are 92.3704%, 42.1193%, 92.6658%, and 75.7839%, respectively. Introduction of 6-gingerol increases average pore size in the hydrogels. Overall, GOZ6 maintains a favorable pore architecture and porosity. This may be beneficial for subsequent cell infiltration, migration, and proliferation. The release behavior of 6-gingerol was subsequently evaluated. As shown in Fig. 3H, under mildly acidic conditions, 6-gingerol is released rapidly during the first 3 d. This release profile indicates that GOZ6 can provide an early burst of anti-inflammatory and antioxidant activity during the initial inflammatory phase of bone repair.

X-ray photoelectron spectroscopy analysis was performed to further verify successful fabrication of the GOZ6 composite hydrogel. In the GOZ6 group, characteristic peaks corresponding to carbon–carbon, carbon–nitrogen, and carbon–oxygen double bonds, calcium, and zinc are clearly detected (Fig. 4A). Two-dimensional correlation FTIR spectroscopy analysis was conducted to investigate molecular interactions among hydrogel components (Fig. 4B and C). Characteristic absorption bands of aldehyde groups and Schiff base-related functional groups are mainly located within the 1,500 to 1,800 wavenumber range. This region was therefore selected for detailed analysis. In the synchronous 2-dimensional FTIR spectrum, a positive cross-peak is observed at approximately 1,550 and 1,683 wavenumbers. This indicates coordinated variation of the associated vibrational modes and suggests molecular interactions between these functional groups. In the asynchronous spectrum, the corresponding positive cross-peak at approximately 1,550 and 1,683 wavenumbers indicates that the spectral change at 1,550 wavenumbers precedes that at 1,683 wavenumbers. The 1,550-wavenumber peak is assigned to nitrogen–hydrogen bending vibration of amino groups. The 1,683-wavenumber peak corresponds to carbon–nitrogen double bond stretching vibration of the Schiff base. These results directly support the formation of Schiff base covalent bonds between oxidized konjac glucomannan and gelatin. In addition, positive cross-peaks at approximately 1,518 and 1,710 wavenumbers are detected in the asynchronous spectrum. The peak at 1,518 wavenumbers is assigned to nitrogen–hydrogen bending vibration following Schiff base formation. The peak at 1,710 wavenumbers corresponds to carbon–oxygen double bond stretching vibration of aldehyde groups. These findings confirm successful oxidation of konjac glucomannan. Collectively, these observations confirm the presence of all designed components in the mixed system. Moreover, the results imply that hydrogen bonding interactions exist between carbon–oxygen double bond groups in the hydrogel matrix and imidazole-associated groups of ZIF-8. 6-Gingerol likely interacts with the hydrogel network through hydrogen bonding involving its phenolic hydroxyl groups. These findings indicate that both composite nanoparticles and 6-gingerol are physically incorporated into the hydrogel matrix.

**Fig. 4.**
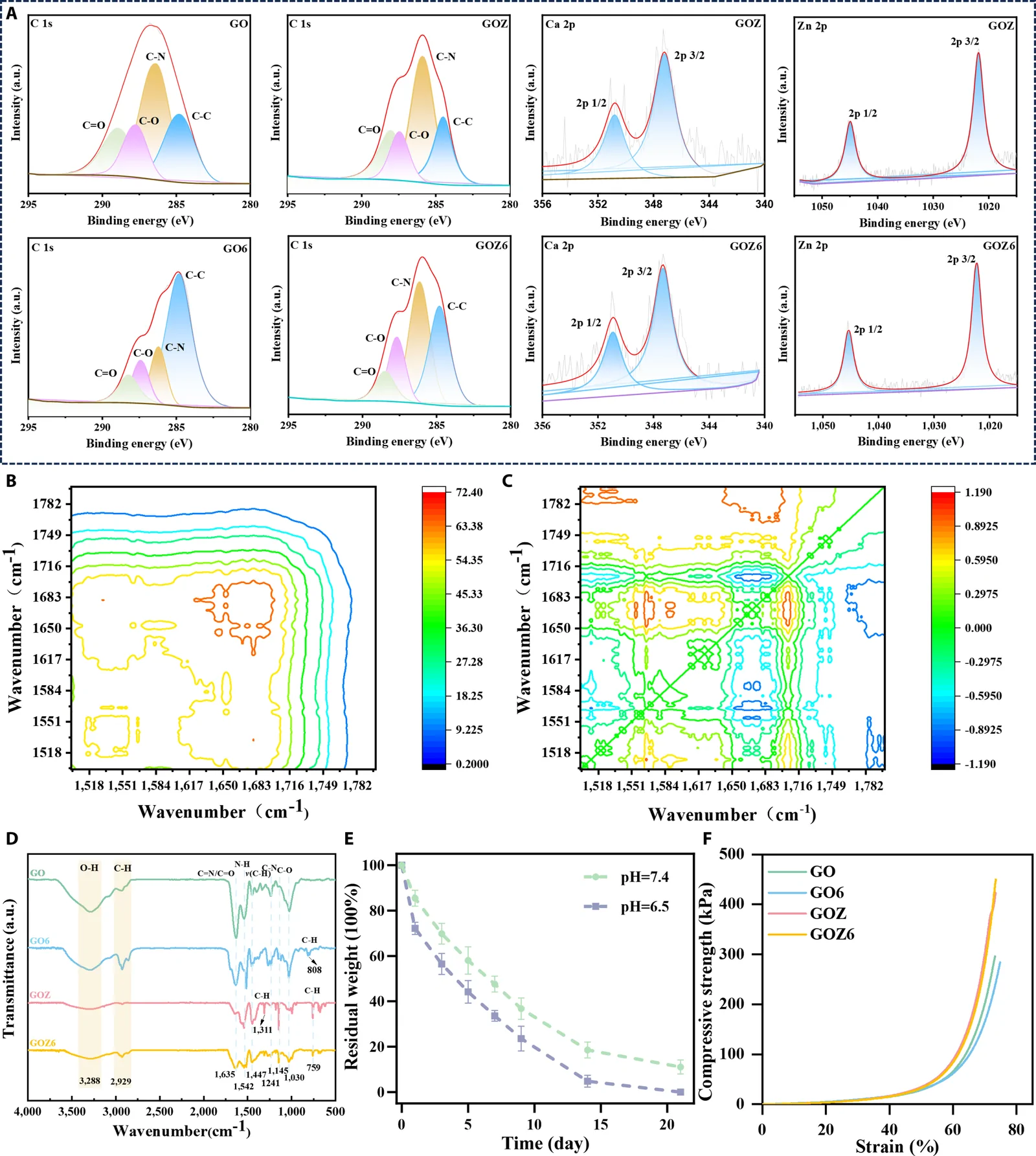
Chemical characterization of GOZ6. (A) XPS spectra acquired for GO, GOZ, GO6, and GOZ6. (B) Synchronous spectra of 2-dimensional infrared detection for each hydrogel group. (C) Asynchronous spectra of 2-dimensional infrared detection for each hydrogel group. (D) FTIR spectra acquired for GO, GOZ, GO6, and GOZ6. (E) Degradation curves of GOZ6 hydrogel under different pH conditions. (F) Stress–strain curves for each group of hydrogels. Data are presented as the mean ± SD (*n* = 3).

Conventional FTIR spectroscopy analysis further supports these conclusions (Fig. 4D). The broad absorption peak at 3,288 wavenumbers corresponds to oxygen–hydrogen stretching vibration. This peak is present in both oxidized konjac glucomannan and 6-gingerol. Following Schiff base formation between gelatin and oxidized konjac glucomannan, oxygen–hydrogen absorption intensity decreases. This indicates rearrangement of hydrogen bonding interactions within the hydrogel network. In addition, ZIF-8 possesses a relatively rigid and microporous structure. Its incorporation may physically disrupt the continuous hydrogen-bonding network formed between long polymer chains of gelatin and oxidized konjac glucomannan, thereby further weakening the oxygen–hydrogen absorption band in the GOZ and GOZ6 groups. The peak at 2,929 wavenumbers, assigned to carbon–hydrogen stretching vibration, shows no marked changes across all groups. The peak at 1,635 wavenumbers corresponds to the carbon–nitrogen double bond of the Schiff base structure. Compared with GO and GO6, absorption intensity at this position is significantly weakened in GOZ and GOZ6. This likely occurs because zinc in ZIF-8 alters the local chemical environment of carbonyl-associated groups through coordination interactions, thereby disturbing their original hydrogen-bonding state. Notably, 6-gingerol also contains carbonyl-related structures. Absorption intensity at this site is therefore partially retained in GOZ6. The peak at 1,542 wavenumbers is attributed to nitrogen–hydrogen vibration of residual gelatin. The peak at 1,447 wavenumbers corresponds to aliphatic carbon–hydrogen bending vibration. The small sharp peak at 1,311 wavenumbers is assigned to in-plane carbon–hydrogen bending of the ZIF-8 ring. In addition, peaks at 1,145 and 1,030 wavenumbers correspond to carbon–oxygen stretching vibration and remain relatively unchanged. Peaks at 808 and 759 wavenumbers in the GO6, GOZ, and GOZ6 groups are assigned to out-of-plane carbon–hydrogen vibrations of the imidazole or aromatic ring. These findings further indicate successful incorporation of composite nanoparticles into the hydrogel.

The oxygen-generation profiles of free CaO₂, GO@CaO₂, and GOZ6 were evaluated (Fig. S11). Free CaO₂ exhibited rapid oxygen generation during the early stage and reached a plateau by day 6, with no further increase thereafter. Incorporation of CaO₂ into the GO hydrogel reduced the initial generation rate and prolonged oxygen production throughout the 14-d observation period. GOZ6 showed the slowest initial oxygen generation but maintained a continuous increase over time. Compared with free CaO₂ and GO@CaO₂, GOZ6 exhibited the most gradual and sustained oxygen-generation profile, consistent with the combined confinement effects of the ZIF-8 framework and hydrogel network. These results indicate that the ZIF-8 coating and hydrogel network jointly restrict the rapid exposure of CaO₂ to the aqueous environment, thereby reducing burst oxygen generation and extending oxygen production in the final hydrogel system.

The degradation behavior of the hydrogels was subsequently evaluated. As shown in Fig. 4E, under mildly acidic conditions, the hydrogel degrades rapidly and is almost completely degraded within approximately 14 d. These results demonstrate that the hydrogel exhibits pH-responsive degradation behavior. This property is favorable for accelerated active component release during the early inflammatory phase. The mechanical properties of the hydrogels were assessed. As shown in Fig. 4F, incorporation of composite nanoparticles enhances hydrogel mechanical performance. This indicates that the inorganic nanocomposite contributes to reinforcement of the hydrogel network. The local retention of the hydrogels was further evaluated under wet conditions (Fig. S12A and B). After equilibration in phosphate-buffered saline (PBS), all 4 hydrogel formulations maintained comparable compressive moduli. In tensile adhesion tests using PBS-pretreated bovine bone, GOZ and GOZ6 exhibited higher wet adhesive strength than did GO and GO6, indicating that incorporation of ZIF-8@CaO₂ enhanced hydrogel adhesion to the wet bone surface, likely through strengthened interfacial interactions and internal network cohesion.

### Cytocompatibility of GOZ6 hydrogels

Excellent cytocompatibility is an essential prerequisite for biomedical application of hydrogel systems. We therefore systematically evaluated the cytotoxicity of the scaffolds toward rat BMSCs and RAW264.7 macrophages (Figs. S13 and S14). Live/dead staining shows that after 48 h of coculture, all groups exhibit high density of viable cells with very few dead cells. Cell viability consistently remains above 90% across all groups. Cell Counting Kit-8 (CCK-8) proliferation assays further confirm that cells in all groups proliferate well on days 1, 3, and 5. The GOZ6 group shows the most favorable proliferation profile. These results demonstrate that the hydrogel system exhibits no apparent cytotoxicity and possesses excellent cytocompatibility.

### GOZ6 regulates macrophage polarization and improves metabolic status

Bone implants must prevent post-implantation immune inflammatory reactions [44]. To investigate the effects of different hydrogel systems on macrophage phenotypic transition, we first examined expression of the M1 marker CD86 and the M2 marker CD206. As shown in Fig. 5A and B, the GO group exhibits the strongest CD86 fluorescence signal. CD86 expression gradually decreases in the GOZ, GO6, and GOZ6 groups, with the lowest level observed in the GOZ6 group. In contrast, CD206 fluorescence is lowest in the GO group. It increases moderately in the GOZ and GO6 groups and reaches the highest level in the GOZ6 group. This indicates that GOZ6 most effectively promotes transition of macrophages from the pro-inflammatory M1 phenotype toward the reparative M2 phenotype.

**Fig. 5.**
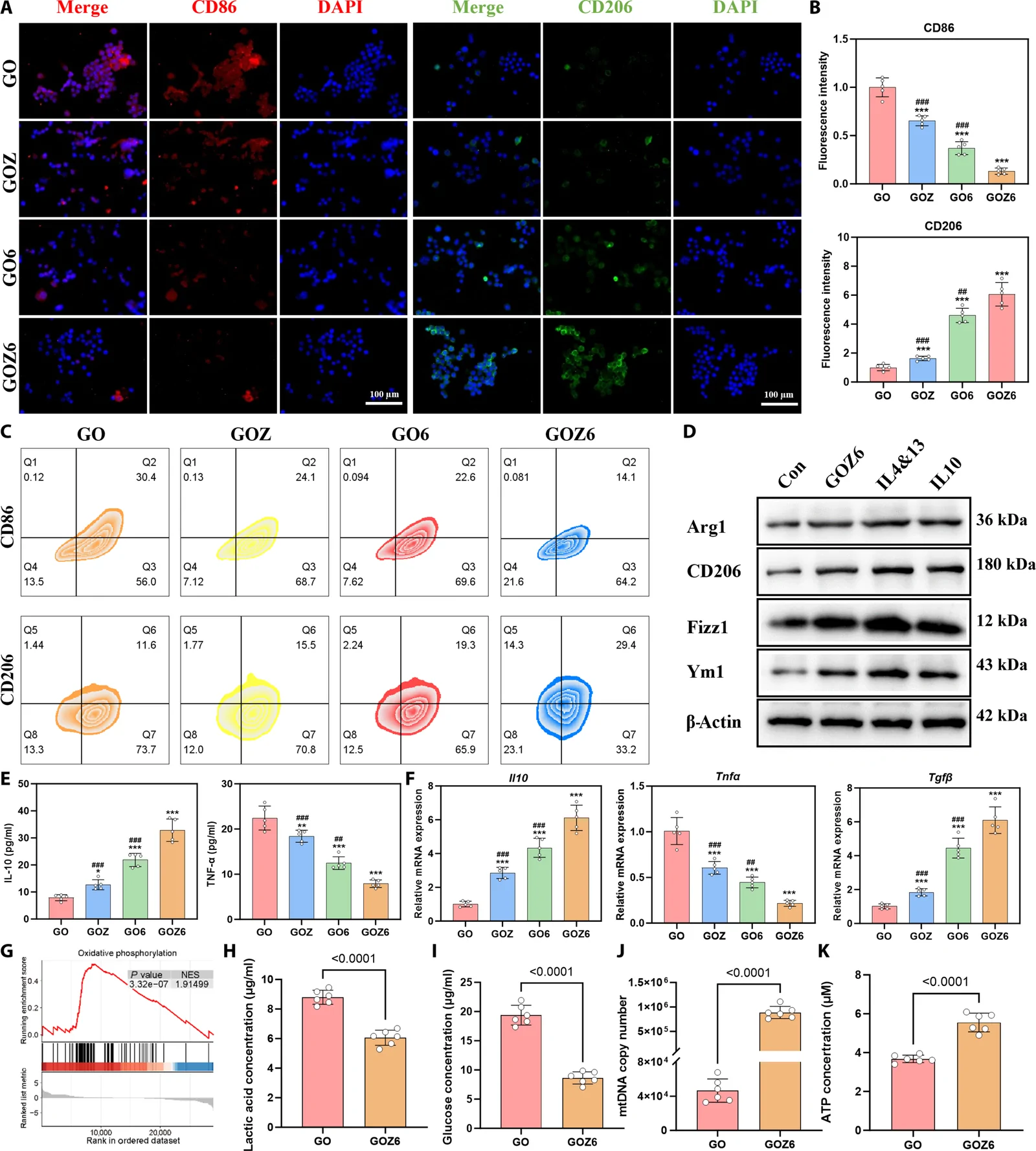
The GOZ6 hydrogel regulates macrophage polarization and reprograms their metabolic phenotype. (A) Representative immunofluorescence images of CD86 and CD206 staining in macrophages from different groups, used to evaluate pro-inflammatory M1 polarization and anti-inflammatory/reparative M2 polarization, respectively. Scale bar, 100 μm. (B) Quantitative analysis of CD86 and CD206 fluorescence intensity. (C) Flow cytometry analysis of macrophages in different groups. (D) Western blot analysis of proteins associated with macrophage polarization. (E) ELISA detection of IL-10 and TNF-α expression levels. (F) RT-qPCR analysis of *IL-10*, *TNF-α*, and *TGF-β* expression in RAW264.7 cells under different treatments. (G) GSEA enrichment analysis of pathways related to oxidative phosphorylation. (H) Detection of lactate levels in RAW264.7 cells under different treatments. (I) Detection of glucose levels in RAW264.7 cells under different treatments. (J) Detection of mtDNA levels in RAW264.7 cells under different treatments. (K) Detection of ATP levels in RAW264.7 cells under different treatments. Data are presented as the mean ± SD (*n* = 5). Compared with the GO group: *P < 0.05, **P < 0.01, ***P < 0.001, ns: not significant (*P* > 0.05). Compared with the GOZ6 group: ^#^*P* < 0.05, ^##^*P* < 0.01, ^###^*P* < 0.001, ns: not significant (*P* > 0.05).

Flow cytometry analysis further confirms this trend (Fig. 5C and Fig. S15). Compared with the GO group, the GOZ6 group shows a markedly reduced proportion of CD86-positive cells and significantly increased proportion of CD206-positive cells. This demonstrates that GOZ6 more effectively suppresses M1-like polarization while promoting M2-like polarization. Western blot analysis further shows that expression levels of M2-associated proteins including Arg1, CD206, Fizz1, and Ym1 are significantly up-regulated in the GOZ6 group (Fig. 5D). This further confirms the ability of this material to drive macrophages toward an anti-inflammatory M2 phenotype.

We next evaluated cytokine secretion and inflammatory gene expression. Enzyme-linked immunosorbent assay (ELISA) analysis showed that GOZ6 markedly altered the cytokine secretion profile of macrophages (Fig. 5E and Fig. S16). Compared with the inflammatory control and other material groups, GOZ6 reduced the secretion of tumor necrosis factor-α (TNF-α), interleukin-12 (IL-12), and interferon-γ (IFN-γ) while increasing IL-10. These changes were consistent with the reduced CD86-positive population, increased CD206-positive population, and corresponding changes in inflammatory and reparative gene expression. Collectively, these results indicate that GOZ6 attenuated the pro-inflammatory macrophage response and promoted a more reparative immune phenotype. Consistently, real-time quantitative polymerase chain reaction (RT-qPCR) analysis reveals that GOZ6 significantly up-regulates expression of anti-inflammatory genes including Il10 and Tgf-β while down-regulating Tnf-α and iNos expression (Fig. 5F and Fig. S17). These findings suggest that GOZ6 not only alters macrophage phenotype but also reshapes their secretory profile toward a microenvironment more favorable for tissue repair.

We further investigated the impact of GOZ6 on macrophage metabolic status. Gene set enrichment analysis (GSEA) shows that oxidative phosphorylation-related pathways are significantly enriched following GOZ6 treatment (Fig. 5G). This suggests a shift from inflammation-associated glycolytic metabolism toward a more mitochondria-dependent oxidative metabolic phenotype. Compared with the GO group, the GOZ6 group exhibits significantly reduced lactate levels and glucose consumption. It also shows markedly elevated adenosine triphosphate (ATP) production and mitochondrial DNA copy number (Fig. 5H to K). These data indicate that GOZ6 suppresses inflammatory metabolic characteristics while improving mitochondrial function and cellular bioenergetic status. This supports macrophage reprogramming toward an M2-like phenotype at the metabolic level. Collectively, GOZ6 demonstrates the strongest capacity to suppress pro-inflammatory polarization, enhance reparative polarization, and restore oxidative metabolism. This indicates its robust immunomodulatory and metabolic reprogramming effects.

### Transcriptomic profiling suggests signaling pathways associated with GOZ6-mediated macrophage reprogramming

To investigate the molecular changes associated with GOZ6-mediated macrophage regulation, transcriptomic analysis was performed in control and GOZ6-treated macrophages. Differential gene expression analysis showed clear separation between the 2 groups (Fig. 6A), indicating that GOZ6 treatment was associated with extensive transcriptional alterations. Kyoto Encyclopedia of Genes and Genomes (KEGG) enrichment analysis further identified changes in multiple immune-related and signal-transduction pathways, with prominent enrichment in the immune system and signal-transduction categories (Fig. 6B). These findings suggest that the effects of GOZ6 on macrophages involve broad transcriptional reprogramming rather than regulation of a single inflammatory mediator.

**Fig. 6.**
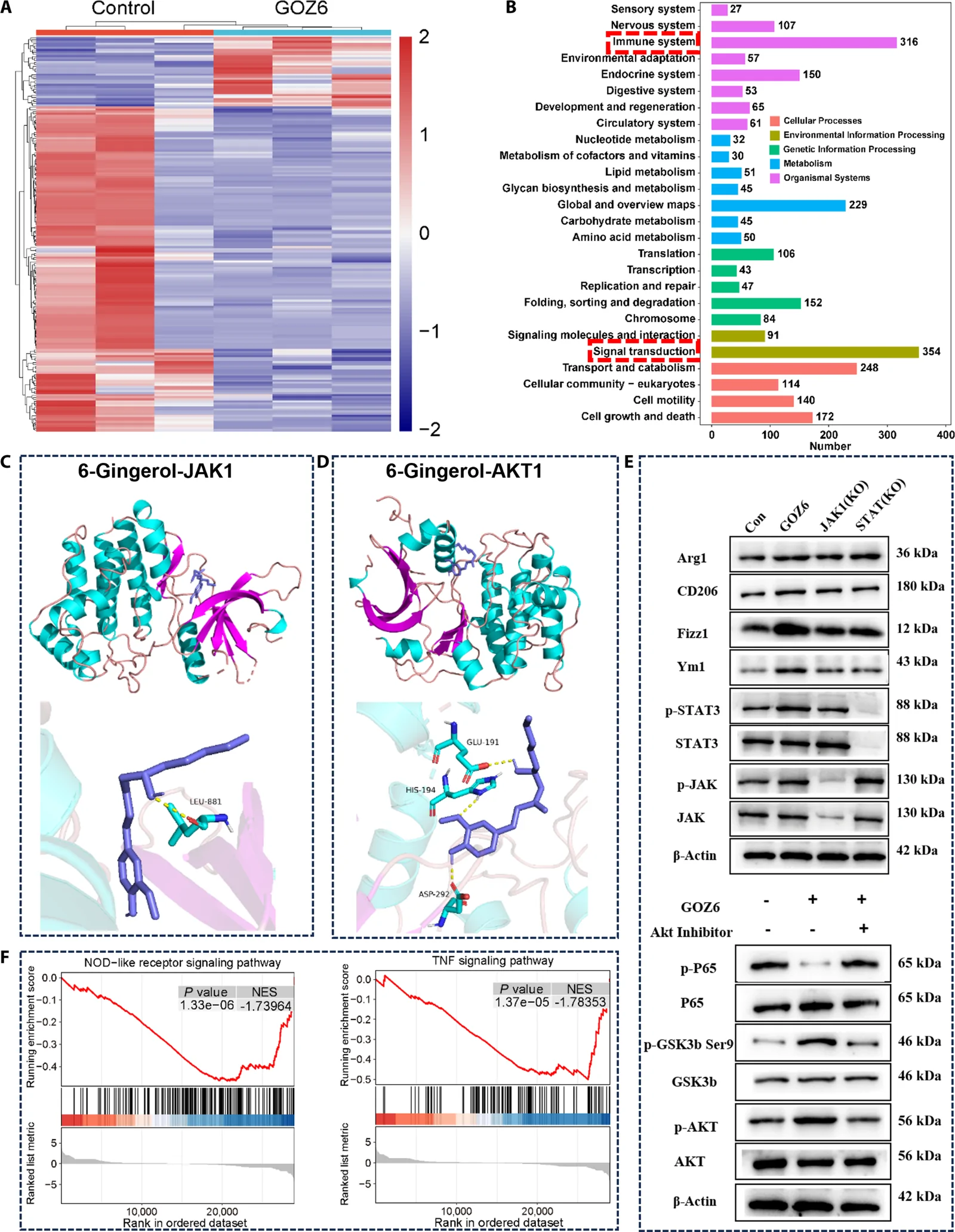
RNA-seq and pathway analysis of GOZ6-treated versus control macrophages. (A) Heatmap of differentially expressed genes between the GOZ6 and Control groups. (B) KEGG pathway enrichment bubble plot based on the differentially expressed genes identified in the GOZ6 versus Control comparison. (C and D) Predicted overall binding conformations and local interactions of 6-gingerol with the candidate proteins AKT1 and JAK1. (E) Representative immunoblotting results showing regulation of the JAK1–STAT3 and PI3K–AKT pathways in RAW264.7 cells under different treatment conditions. (F) Representative GSEA enrichment plots of key pathways, including the NOD-like receptor signaling pathway and TNF signaling pathway.

Based on the transcriptomic and pathway analyses, molecular docking was used as a hypothesis-generating screening approach to evaluate the predicted structural compatibility of 6-gingerol with selected candidate signaling proteins. The predicted binding energies of 6-gingerol with AKT1 and Janus kinase 1 (JAK1) were −7.4 and −8.9 kcal mol^−1^, respectively. The predicted docking poses suggested possible hydrogen-bond interactions with Glu^191^, His^194^, and Asp^292^ of AKT1 and with Leu^881^ of JAK1 (Fig. 6C and D). These computational predictions were used to prioritize candidate signaling axes for downstream functional examination and were not interpreted as standalone evidence of direct binding or intracellular target engagement.

GOZ6 treatment increased IL-10 expression in macrophages and was accompanied by changes in JAK1–signal transducer and activator of transcription 3 (STAT3)-associated signaling (Fig. S18 and Fig. 6E). Genetic disruption of JAK1 or STAT3 attenuated the GOZ6-induced increase in the reparative macrophage phenotype, supporting the functional contribution of this signaling pathway to GOZ6-mediated macrophage regulation. However, these findings do not establish JAK1 as a direct molecular target of 6-gingerol.

GOZ6 treatment was also associated with increased AKT phosphorylation and corresponding changes in the phosphorylation of downstream glycogen synthase kinase 3β (GSK3β) and nuclear factor κB (NF-κB) p65 under M2-polarizing conditions. Pharmacological inhibition of AKT reduced GOZ6-induced AKT phosphorylation and partially reversed the associated changes in downstream signaling. These findings support the involvement of AKT-associated signaling in the immunomodulatory effects of GOZ6 but do not demonstrate direct targeting of AKT1 by 6-gingerol. GSEA showed positive enrichment of oxidative phosphorylation in the GOZ6 group, whereas the NOD (nucleotide-binding oligomerization domain)-like receptor signaling pathway, TNF signaling pathway, and fatty acid biosynthesis were negatively enriched (Fig. 6F). These transcriptomic changes were consistent with enhanced oxidative metabolism and suppression of pro-inflammatory signaling programs.

Collectively, the results support the functional involvement of JAK1–STAT3- and phosphatidylinositol 3-kinase (PI3K)–AKT-associated signaling in GOZ6-mediated macrophage reprogramming. Molecular docking served only as a hypothesis-generating tool for candidate selection. Accordingly, these signaling axes are interpreted as pathways participating in the immunomodulatory effects of GOZ6 rather than as confirmed direct intracellular targets of 6-gingerol.

### GOZ6 treatment is associated with enhanced endothelial angiogenic behavior

Macrophage polarization profoundly influences vascular regeneration. We therefore used a macrophage–endothelial cell coculture system to evaluate the effects of different treatments on angiogenesis (Fig. 7A). As shown in Fig. 7B and C, both the GOZ and GO6 groups promote human umbilical vein endothelial cell (HUVEC) migration to some extent compared with the GO group. The greater response observed in the GOZ group may reflect the combined effects of oxygen generation and bioactive ion release. The GOZ6 group shows the highest number of migrated cells, indicating the strongest endothelial recruitment ability.

**Fig. 7.**
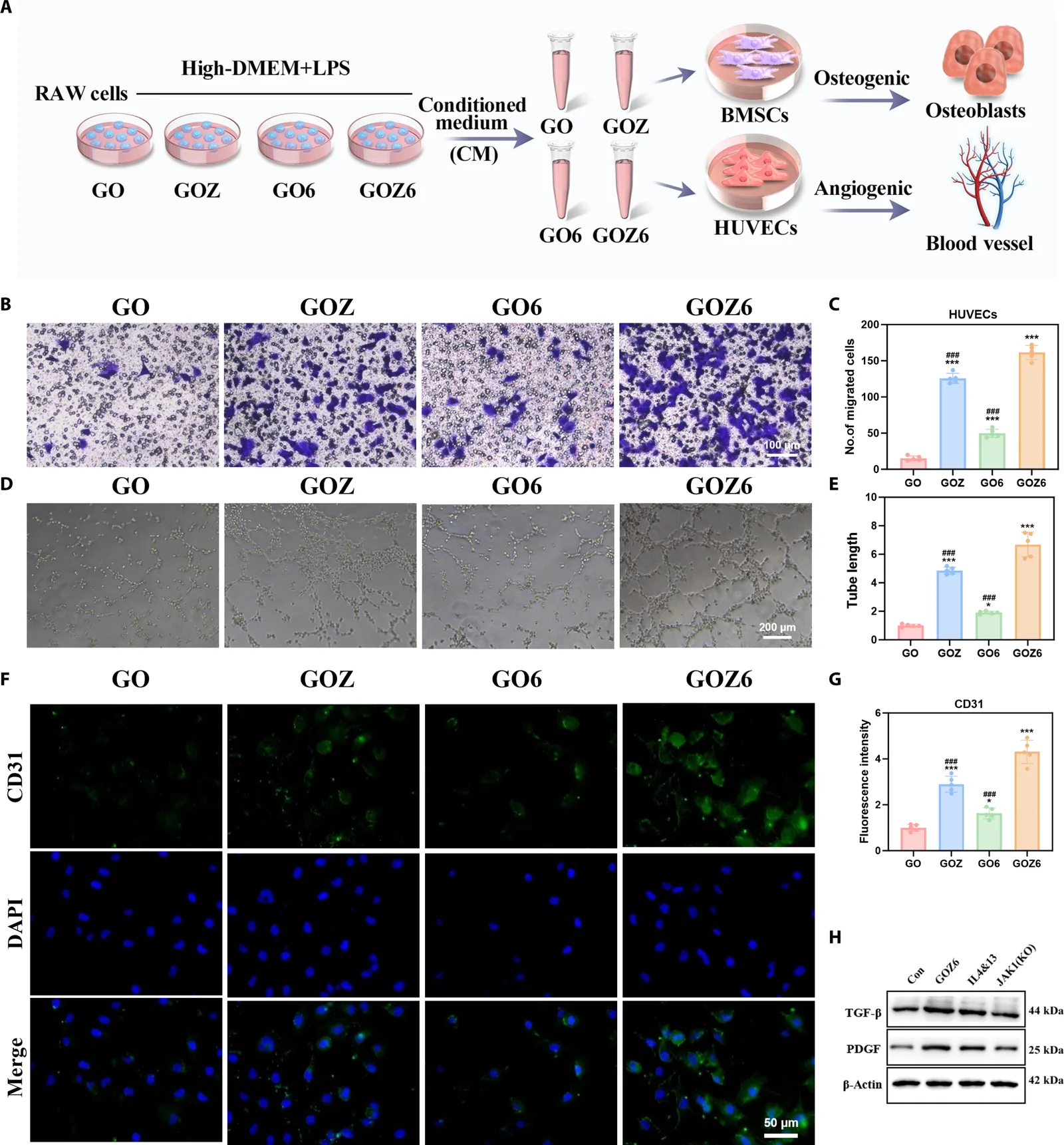
GOZ6 promotes angiogenesis through macrophage–endothelial cell crosstalk. (A) Schematic illustration of the experimental setup. (B) Representative images of the endothelial cell migration assay under different treatment conditions. Scale bar, 100 μm. (C) Quantitative analysis of the number of migrated cells. (D) Representative images of the in vitro tube formation assay of endothelial cells. Scale bar, 200 μm. (E) Quantitative analysis of tube formation capacity. (F) Representative immunofluorescence images of CD31 staining for evaluation of the angiogenesis-related phenotype. Scale bar, 50 μm. (G) Quantitative analysis of CD31 fluorescence intensity. (H) Representative immunoblotting results showing the regulation of angiogenesis-related proteins in RAW264.7 cells under different treatment conditions. Data are presented as the mean ± SD (*n* = 5). Compared with the GO group: *P < 0.05, **P < 0.01, ***P < 0.001, ns: not significant (*P* > 0.05). Compared with the GOZ6 group: ^#^*P* < 0.05, ^##^*P* < 0.01, ^###^*P* < 0.001, ns: not significant (*P* > 0.05).

In vitro tube formation assays further show that the GOZ6 group forms more complete and continuous capillary-like networks. Total tube length is significantly greater in the GOZ6 group than in all other groups. This demonstrates that GOZ6 markedly enhances the angiogenic behavior of endothelial cells (Fig. 7D and E). Consistently, CD31 immunofluorescence staining reveals the strongest green fluorescence signal in the GOZ6 group (Fig. 7F). Quantitative analysis confirms that CD31 expression is highest in this group, further supporting its superior pro-angiogenic activity (Fig. 7G).

To further characterize the regenerative phenotype of GOZ6-treated macrophages, the expression of several regeneration-associated proteins was examined. Western blot analysis showed increased transforming growth factor-β (TGF-β) and platelet-derived growth factor (PDGF) expression in RAW264.7 cells following GOZ6 treatment (Fig. 7H). These changes suggest that GOZ6 promotes a macrophage phenotype with enhanced reparative potential, which may contribute to a microenvironment favorable for vascular regeneration. Collectively, the direct stimulation and conditioned-medium experiments suggest that GOZ6 supports endothelial angiogenic behavior through integrated material-mediated and macrophage-associated microenvironmental regulation.

### GOZ6 alleviates oxidative stress, improves mitochondrial function, and promotes osteogenic differentiation of BMSCs

In the diabetic inflammatory and oxidative stress microenvironment, mitochondrial dysfunction and impaired osteogenic capacity of BMSCs represent major barriers to bone regeneration. We therefore further evaluated the effects of GOZ6 on oxidative stress, mitochondrial function, and osteogenic differentiation in these cells.

As shown in Fig. 8A and Fig. S19, GOZ6 increased red aggregate fluorescence while reducing green monomer fluorescence. This suggests improved mitochondrial membrane potential, which may be partly attributed to the antioxidant activity of 6-gingerol. Flow cytometric analysis of JC-1 further confirms that the proportion of high-membrane-potential cells is significantly increased in the GOZ6 group (Fig. 8B and Fig. S20). This indicates that GOZ6 effectively improves mitochondrial functional status and maintains mitochondrial membrane potential stability.

**Fig. 8.**
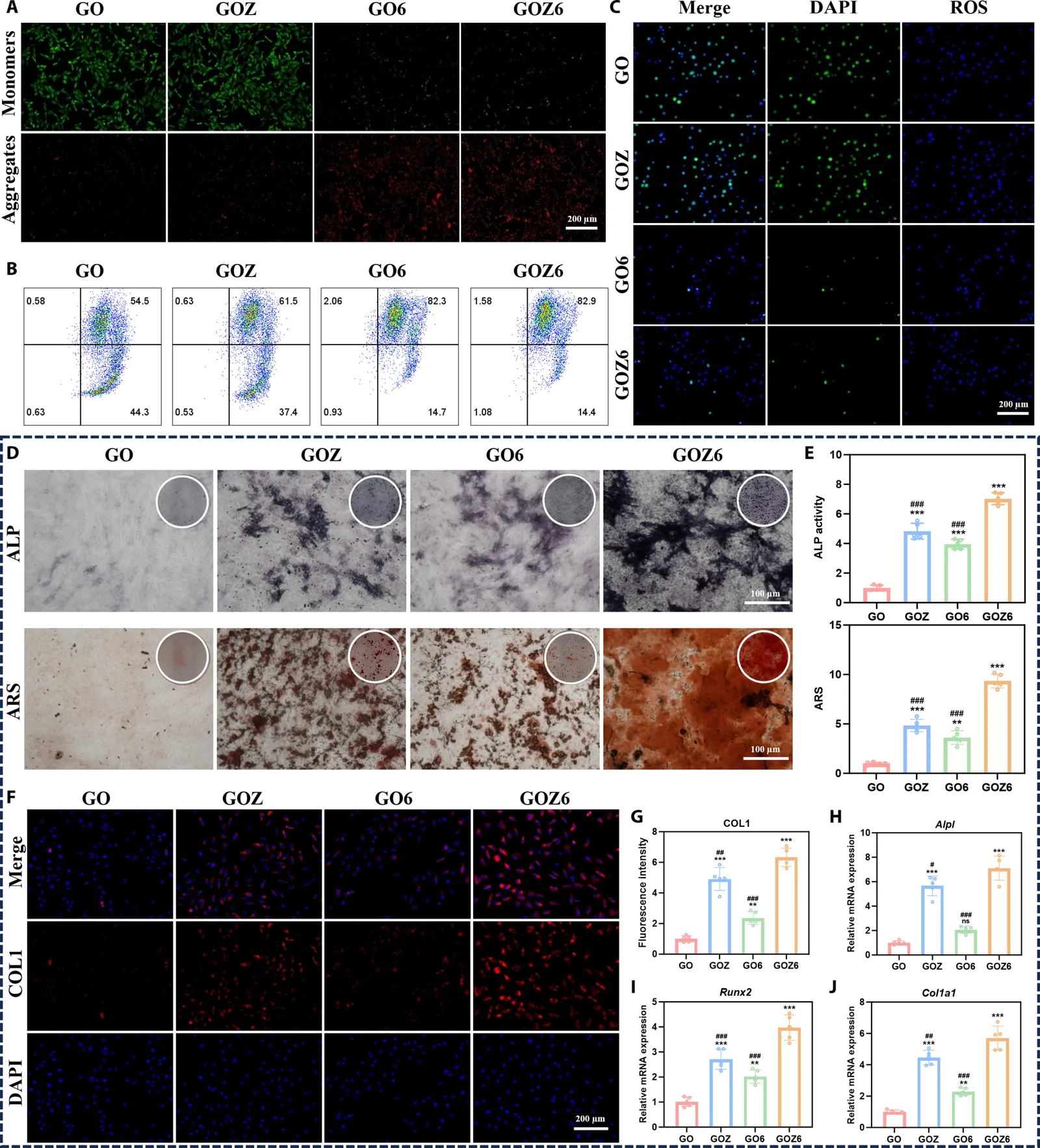
GOZ6 improves mitochondrial function in BMSCs and promotes osteogenic differentiation. (A) Representative fluorescence images of JC-1 in BMSCs under different hydrogel treatments. Scale bar, 200 μm. (B) JC-1 flow cytometric analysis of BMSCs in different groups. (C) Representative fluorescence images of ROS staining in BMSCs under different hydrogel treatments. (D) Representative images of alkaline phosphatase (ALP) staining and Alizarin Red S (ARS) staining. Scale bar, 100 μm. (E) Quantitative analysis of ALP activity and mineralized nodule formation. (F) Immunofluorescent staining of BMSCs cultured for 3 d: green (COL1), red (phalloidin), and blue (DAPI). Scale bar, 200 μm. (G) Quantitative analysis of COL1 fluorescence intensity. (H to J) RT-qPCR analysis of osteogenesis-related gene expression (*Alpl, Runx2,* and *Col1a1*) in BMSCs. Data are presented as the mean ± SD (*n* = 5). Compared with the GO group: *P < 0.05, **P < 0.01, ***P < 0.001, ns: not significant (*P* > 0.05). Compared with the GOZ6 group: ^#^*P* < 0.05, ^##^*P* < 0.01, ^###^*P* < 0.001, ns: not significant (*P* > 0.05).

Reactive oxygen species (ROS) staining results show that intracellular ROS levels are relatively high in the GO and GOZ groups (Fig. 8C and Fig. S21). Under normal conditions, burst release of calcium peroxide could potentially increase ROS generation. However, the ZIF-8 structure restricts abrupt release, and Zn^2+^ release may support cellular antioxidant defenses and redox regulation. Accordingly, ROS levels in the GO and GOZ groups are comparable. In contrast, ROS levels are markedly reduced in the GO6 and GOZ6 groups. This indicates that 6-gingerol-containing systems are more effective at alleviating oxidative stress. Flow cytometric analysis of ROS further supports this result (Fig. S22A and B).

We next assessed osteogenic differentiation. Alkaline phosphatase (ALP) and Alizarin Red S (ARS) staining show that the GOZ group exhibits stronger staining intensity and larger mineralized area than does the GO6 group. This is likely due to the direct osteoregulatory effect of calcium ions released from GOZ. Among all groups, the GOZ6 group shows the strongest staining and largest positive area (Fig. 8D). Quantitative analysis further confirms that both ALP activity and mineralization are significantly higher in the GOZ6 group than in the GO, GOZ, and GO6 groups (Fig. 8E). These results indicate that GOZ6 not only promotes early osteogenic differentiation of BMSCs but also significantly enhances late-stage mineral deposition.

Further immunofluorescence staining shows that COL1 expression is markedly increased in the GOZ6 group (Fig. 8F and G). This suggests enhanced bone matrix protein synthesis. Consistently, RT-qPCR analysis reveals that mRNA levels of key osteogenic genes including *Alpl*, *Runx2*, *Col1a1*, and *Bglap* are significantly up-regulated in the GOZ6 group (Fig. 8H to J and Fig. S23). Notably, both GOZ and GO6 alone improve osteogenic indices to some extent. GOZ shows a relatively stronger effect. However, the combined GOZ6 system produces the greatest enhancement. This indicates cooperative effects between oxygen and ion regulation and 6-gingerol-mediated antioxidative protection. Collectively, GOZ6 effectively alleviates oxidative stress in BMSCs, restores mitochondrial function, and significantly promotes osteogenic differentiation.

### GOZ6 significantly promotes diabetic calvarial defect repair and improves the local immune-angiogenic-osteogenic microenvironment

Finally, we evaluated the in vivo therapeutic efficacy of GOZ6 in a diabetic rat calvarial defect model containing 5 groups: Blank control, GO, GOZ, GO6, and GOZ6 (Fig. 9A). In the Blank control group, defects were left untreated without material implantation. Micro-computed tomography (CT) reconstruction showed limited spontaneous bone regeneration in the Blank control and GO group at both 4 and 8 weeks. In contrast, GOZ and GOZ6 promoted more evident new bone formation, with GOZ6 showing the greatest defect filling and the smallest residual defect area (Fig. 9B). Quantitative analysis further demonstrated that bone mineral density (BMD) and bone volume fraction (BV/TV) were significantly higher in the GOZ6 group than in the Blank control, GO, GO6, and GOZ groups at both time points, with a more pronounced difference at 8 weeks (Fig. 9C). These results indicate that the integrated oxygen, ion, and immunoregulatory functions of GOZ6 substantially enhance bone regeneration beyond spontaneous healing or treatment with the hydrogel matrix alone.

**Fig. 9.**
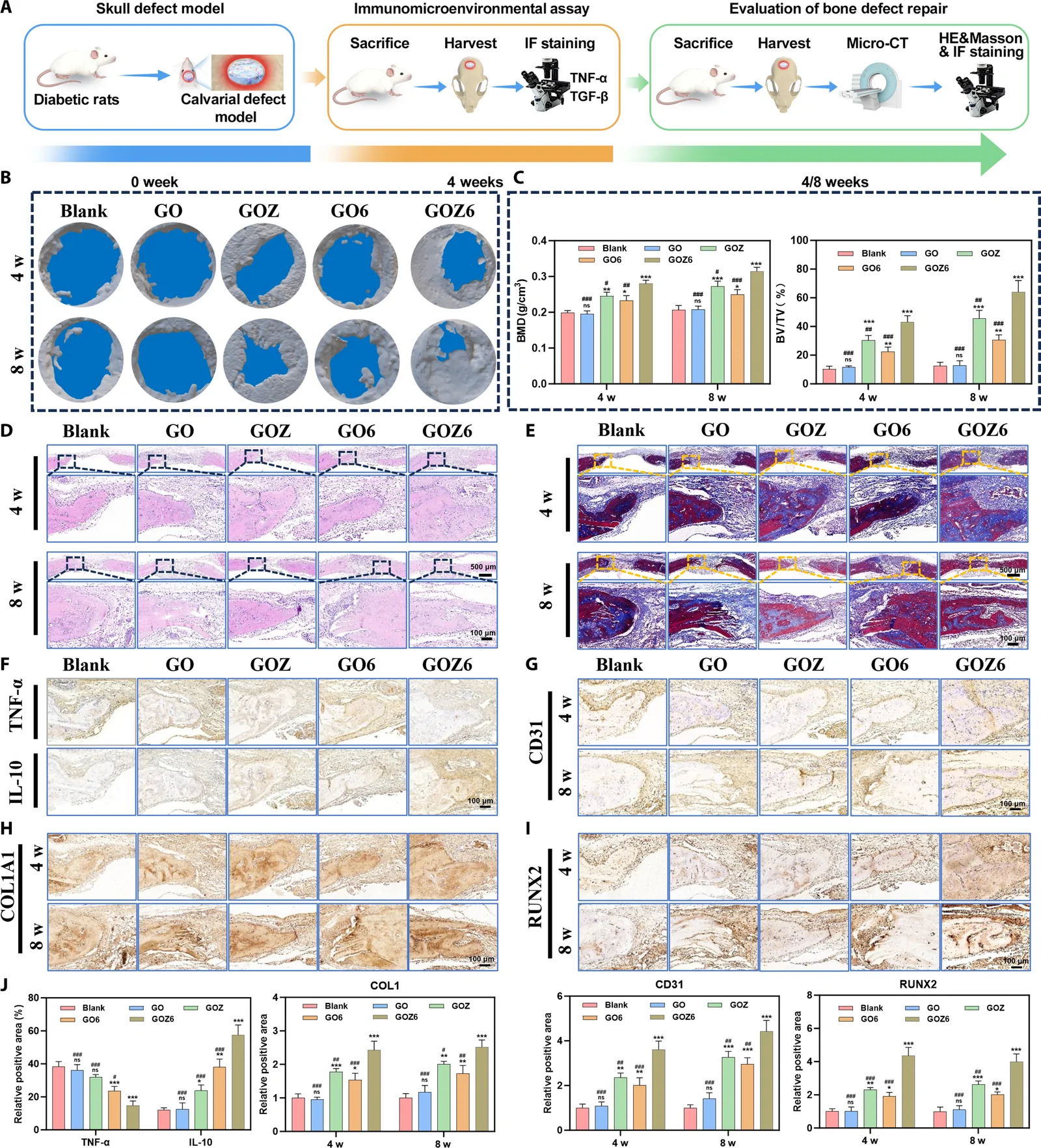
In vivo evaluation of bone regeneration in a diabetic rat calvarial defect model. (A) Schematic illustration of the modeling process and animal experimental procedures. (B) Micro-CT scanning and reconstructed 3-dimensional images of calvarial bone defects implanted with different hydrogel. (C) Quantitative evaluation of bone regeneration in the defect region based on bone mineral density (BMD) and bone volume fraction (BV/TV). (D and E) H&E and Masson’s trichrome staining of newly formed bone at 4 and 8 weeks after hydrogel implantation. (F) Representative immunohistochemical images of TNF-α and IL-10 staining. (G) Representative immunohistochemical images of CD31 staining. (H) Representative immunohistochemical images of COL1A1 staining. (I) Representative immunohistochemical images of RUNX2 staining. (J) Quantitative analysis of histological staining and immunohistochemical results. Data are presented as the mean ± SD (*n* = 5 animals per group). Compared with the GO group: *P < 0.05, **P < 0.01, ***P < 0.001, ns: not significant (*P* > 0.05). Compared with the GOZ6 group: ^#^*P* < 0.05, ^##^*P* < 0.01, ^###^*P* < 0.001, ns: not significant (*P* > 0.05).

Histological findings were consistent with the micro-CT results. The Blank control and GO groups retained large unrepaired regions at both time points, whereas GOZ6 promoted greater tissue filling at week 4 and more continuous and mature new bone formation by week 8 (Fig. 9D). Masson’s trichrome staining further showed markedly increased collagen deposition and denser tissue organization in the GOZ6 group compared with the Blank control and other treatment groups (Fig. 9E).

Immunohistochemical analysis further demonstrated that GOZ6 significantly remodeled the local regenerative microenvironment. Compared with the Blank control and all other groups, GOZ6 reduced TNF-α expression and increased IL-10 expression, indicating attenuation of local inflammation (Fig. 9F). GOZ6 also enhanced COL1A1 expression and produced the strongest CD31 and RUNX2 staining at both 4 and 8 weeks, reflecting improved matrix deposition, vascularization, and osteogenic differentiation (Fig. 9G to I). Quantitative analysis of the histological and immunohistochemical results further confirmed the superior regenerative performance of GOZ6 over the Blank control and hydrogel matrix control (Fig. 9J).

To evaluate systemic biosafety, major organs including heart, liver, spleen, lung, and kidney were collected at 8 weeks after hydrogel implantation (Fig. S24). Histological examination shows no obvious pathological abnormalities or toxic reactions in any organ. This further supports the favorable in vivo biocompatibility of the hydrogel system. Overall, GOZ6 significantly improves the local inflammatory state, enhances angiogenesis and bone matrix deposition, and ultimately promotes higher-quality new bone formation in diabetic calvarial defects. This demonstrates its excellent bone repair capacity and good biosafety in vivo.

## Discussion

Diabetic bone repair is impaired by persistent inflammation, oxidative stress, hypoxia, inadequate vascular regeneration, and metabolic disturbance. Hyperglycemia and AGEs further aggravate these abnormalities and compromise skeletal healing [45,46]. Macrophages fail to transition in a timely manner from an inflammation-associated state to a repair-associated state. The relationship between vascular regeneration and osteogenesis is impaired [47]. Mitochondrial function and differentiation potential of mesenchymal stem cells are also severely restricted. These observations suggest that diabetic bone defects may require interventions targeting several pathological processes rather than a single factor [48].

Oxygen plays a crucial role in the bone repair process. Previous studies have shown that calcium peroxide-based oxygen-generating materials can improve local hypoxia and promote angiogenesis and bone regeneration [49]. However, when pristine calcium peroxide is directly exposed to aqueous environments, it tends to decompose rapidly, resulting in burst oxygen release, hydrogen peroxide accumulation, and local alkalization. These effects may further increase oxidative stress, impair mitochondrial function, and weaken regenerative efficacy [50,51]. Controlled oxygen generation is therefore needed to relieve hypoxia without increasing oxidative stress or causing marked local pH changes. To address this limitation, ZIF-8@CaO₂ was designed to combine the oxygen and Ca^2+^ releasing ability of CaO₂ with the pH-responsive porous confinement of ZIF-8, thereby reducing the uncontrolled decomposition of pristine CaO₂ and enabling more sustained oxygen and ion delivery [36]. In this system, ZIF-8 encapsulation slowed CaO₂ exposure and reduced abrupt peroxide-related changes under mildly acidic conditions.

In addition to oxygen regulation, macrophages are also critical for bone regeneration [52]. However, previous studies have focused mainly on the apparent outcomes of suppressing inflammatory cytokines or promoting phenotypic conversion. Comparatively less attention has been paid to changes in macrophage metabolic state [53]. Recent advances in immunometabolism suggest that differences between inflammation-associated macrophages and repair-associated macrophages are reflected not only in cytokine profiles but also in energy metabolism and mitochondrial function [54]. If the underlying metabolic basis is not altered, transient reduction of inflammatory mediators may not be sufficient to maintain a stable repair-associated state [55]. In this study, results show that macrophage phenotypic transition and metabolic remodeling occur simultaneously after GOZ6 treatment. These results indicate that GOZ6 altered both macrophage phenotype and metabolic activity.

Pathway analyses provided additional evidence for these macrophage changes. Although 6-gingerol is known to possess antioxidant and anti-inflammatory properties [56], its role in macrophage signaling regulation within the bone regenerative microenvironment has remained unclear. Previous studies have shown that 6-gingerol can increase IL-10 expression [57]. IL-10 is a key anti-inflammatory factor that regulates innate immunity and inflammatory responses. It exerts its effects by enhancing mitochondrial respiration and inhibiting inflammasome activation. This mechanism relies on the IL-10 receptor–STAT3 signaling axis. Disruption of this pathway can trigger excessive inflammatory responses [58]. In this study, GOZ6 increased IL-10 expression and JAK1/STAT3 phosphorylation. At the same time, changes in AKT, GSK3β, and p65 indicate that GOZ6 may also suppress inflammatory transcriptional output through the PI3K–AKT-related pathway. This is consistent with evidence that macrophage polarization is regulated by multiple signaling pathways rather than a single pathway [59]. In the present study, pathway-perturbation experiments support the involvement of JAK1–STAT3 and PI3K–AKT-associated signaling in the overall immunomodulatory effects of GOZ6. However, molecular docking only suggests possible interactions between 6-gingerol and JAK1 or AKT1 and does not establish direct binding or demonstrate that these pathways are regulated solely by 6-gingerol. The observed macrophage responses likely reflect the combined effects of the GOZ6 components rather than the action of 6-gingerol alone. We also considered whether oxygen release might affect 6-gingerol activity. Because CaO₂ was confined by ZIF-8 and further embedded within the hydrogel network, ZIF-8 encapsulation and hydrogel confinement reduced the rapid exposure of CaO₂ and can limit persistent H₂O₂ accumulation, which may lower the risk of peroxide-related oxidative stress. The preserved antioxidant and immunomodulatory effects observed in GOZ6-treated cells suggest that oxygen release did not abolish 6-gingerol function under the present conditions.

Under mildly acidic conditions, GOZ6 released 6-gingerol relatively rapidly, while oxygen generation and Zn and Ca release were more sustained. The early release of 6-gingerol was associated with reduced oxidative stress and inflammatory macrophage markers, whereas the prolonged oxygen and ion release occurred alongside enhanced endothelial and osteogenic responses. These processes were not temporally isolated, and the present data do not establish a strict sequential mechanism. Nevertheless, the different release profiles provide a possible explanation for the broader biological effects observed with GOZ6 compared with GO6 or GOZ alone.

Osteogenic impairment in diabetic bone regeneration should not be interpreted simply as a lack of osteogenic factors. Elevated oxidative stress and mitochondrial dysfunction in the diabetic environment directly compromise the differentiation potential and matrix synthesis capacity of stem cells [60]. Direct promotion of osteogenesis by osteogenic factors or inorganic ions alone may be insufficient if cells remain under high oxidative pressure and low-energy conditions [61]. In our research, GOZ6 reduced oxidative stress and improved mitochondrial membrane potential while increasing osteogenic markers, suggesting that redox regulation contributed to the osteogenic response. GO showed limited effects, whereas GO6 and GOZ produced partial improvements and GOZ6 produced the strongest responses. These group differences indicate contributions from both 6-gingerol and ZIF-8@CaO₂.

Recent studies have increasingly emphasized that diabetic bone regeneration requires integrated regulation of the pathological microenvironment. For example, spatiotemporal immunomodulatory membranes have been developed to coordinate osteo-vascular regeneration in diabetic periosteal defects, while functional dynamic hydrogels and nanoenzyme-based composite hydrogels have been reported to regulate inflammation, oxidative stress, and osteogenesis in diabetic bone defects [7,9,18,22]. GOZ6 combines pH-responsive oxygen generation, ion release, antioxidant protection, and macrophage regulation in an injectable polysaccharide hydrogel. These results suggest that simultaneously addressing hypoxia, inflammation, oxidative stress, and impaired osteogenesis may be advantageous over targeting only one of these processes.

The dynamic characteristics of GOZ6 itself are an important prerequisite for the performance of this system. Responsive biomaterials can adjust their release behavior according to changes in the local microenvironment [62]. In bone repair, scaffold structure and mechanical compatibility are also important for tissue reconstruction [63,64]. Compared with rigid metallic scaffolds, GOZ6 was designed as an injectable hydrogel to adapt to irregular diabetic bone defects while providing local microenvironmental regulation. The biological priorities of diabetic bone defect repair differ across stages. The early stage requires control of inflammation, oxidative stress, and hypoxia. Later stages depend more on vascular stabilization, matrix deposition, and mineralization [48,65]. Through its dynamic Schiff base network, GOZ6 achieves pH-responsive release. Although matrix metalloproteinases (MMPs) are zinc-dependent proteases and may contribute to degradation of the gelatin component, increased extracellular Zn^2+^ release does not necessarily lead to direct MMP activation because MMP activity is regulated by enzyme activation, endogenous inhibitors, and local inflammatory signals. Therefore, GOZ6 degradation is considered to be primarily driven by acid-sensitive Schiff base hydrolysis, while a possible contribution of Zn^2+^-associated MMP regulation requires further validation using MMP activity assays and specific inhibitors.

GOZ6 was designed to address hypoxia, inflammation, oxidative stress, and impaired vascular and bone regeneration within the same treatment. GOZ6 combines injectable defect filling with local delivery of bioactive components. However, its performance was not directly compared with autologous bone or commercially available calcium phosphate grafts in the present study. The long-term degradation, biosafety, component stability, and direct molecular interactions of 6-gingerol require further investigation. In addition, the manufacturing process and key quality attributes of GOZ6 should be standardized, and its therapeutic performance should be further validated in large-animal load-bearing bone defect models with comparison against commercial bone graft substitutes.

Overall, this study suggests that repair of diabetic bone defects depends more on combined regulation of inflammation, oxidative stress, hypoxia, and osteogenic impairment than on amplification of any single stimulus. Direct exposure of CaO₂ to aqueous environments may cause rapid oxygen generation and peroxide-related changes. In GOZ6, ZIF-8 encapsulation and hydrogel confinement moderated CaO₂ exposure and limited persistent H₂O₂ accumulation. GOZ6 reduced local inflammation, improved cellular metabolic and mitochondrial function, and enhanced vascularized bone formation in the diabetic defect model.

## Conclusion

In summary, we developed a pH-responsive polysaccharide hydrogel, GOZ6, for diabetic bone regeneration by integrating ZIF-8@CaO₂-mediated oxygen/calcium delivery with 6-gingerol-mediated antioxidative and immunomodulatory functions. GOZ6 exhibited rapid gelation, porous architecture, pH-responsive degradation, sustained oxygen release, and good cytocompatibility. In vitro, GOZ6 promoted reparative macrophage polarization, improved macrophage oxidative metabolism, enhanced endothelial angiogenic activity, reduced oxidative stress in BMSCs, and promoted osteogenic differentiation, with these immunometabolic effects being associated with JAK1–STAT3 and PI3K–AKT signaling. In vivo, GOZ6 enhanced vascularized bone formation, collagen deposition, and osteogenic marker expression in diabetic calvarial defects while reducing local inflammation and showing favorable biosafety. These findings suggest that GOZ6 is a promising multifunctional hydrogel platform for remodeling the diabetic bone defect microenvironment.

## Methods

### Preparation of GOZ6

Three grams of konjac glucomannan was dissolved in 100 ml of reverse-osmosis water. The mixture was heated and stirred at 60 °C for full dissolution, followed by cooling to 40 °C. A total of 15ml of 0.1 g/ml sodium periodate solution was added dropwise, and the reaction was maintained at 40 °C for 3 h. Subsequently, 4.0 ml of ethylene glycol was added, and the mixture was stirred for 10 min to terminate the oxidation reaction. The resulting solution was transferred to a dialysis bag (molecular weight cutoff: 14,000 Da) and dialyzed against reverse-osmosis water for 7 d with water renewal twice daily. The dialyzed solution was finally freeze-dried to yield oxidized konjac glucomannan.

Ten grams of calcium chloride was dissolved in 50 ml of reverse-osmosis water, and the resulting solution was poured into 600 ml of anhydrous ethanol, followed by stirring for 10 min. Fifty milliliters of 30% hydrogen peroxide was added, and the mixture was stirred for 2 min. Subsequently, 50 ml of 1M ammonia solution was added dropwise. After 30 min of stirring, the mixture was centrifuged at 8,000 rpm for 3 min, and the solid was washed three times with anhydrous ethanol. The solid was freeze-dried to yield calcium peroxide powder. Zinc nitrate hexahydrate (0.146 g) was dissolved in 5 ml of methanol. 2-Methylimidazole (0.325 g) was dissolved in another 5 ml of methanol, into which 100 mg of the as-prepared calcium peroxide powder was incorporated. The two solutions were combined and stirred at 25 °C for 3 h. The mixture was centrifuged at 8,000 rpm for 3 min, and the product was washed three times with anhydrous ethanol. Finally, the product was freeze-dried to obtain ZIF-8@CaO₂ powder.

A total of 5 ml of 5% w/v oxidized konjac glucomannan solution and 5 ml of 15% w/v gelatin solution were prepared. The two solutions were mixed thoroughly, and the pH value of the mixture was adjusted to 8.0. The resulting mixture was subsequently poured into molds to fabricate the GO hydrogel. For the preparation of the GO6 hydrogel, 5 mg of 6-gingerol was incorporated into the gelatin solution in advance, followed by the same preparation procedures. For the GOZ hydrogel, 100 mg of ZIF-8@CaO₂ was added to the gelatin solution prior to subsequent operations with identical steps. For the GOZ6 hydrogel, both 100 mg of ZIF-8@CaO₂ and 5 mg of 6-gingerol were supplemented into the gelatin solution, and the hydrogel was fabricated following the same protocol.

### Characterization of nanoparticles and GOZ6 hydrogels

The particle size and polydispersity of CaO₂, ZIF-8, and ZIF-8@CaO₂ were measured by dynamic light scattering. Their morphology and elemental distribution were characterized by SEM/TEM and EDS. SEM imaging was performed using a Scios 2 DualBeam FIB-SEM system (Thermo Fisher Scientific, USA). The crystalline structure, surface chemistry, and functional groups were analyzed by XRD, x-ray photoelectron spectroscopy (XPS) (AXIS Ultra DLD, Kratos Analytical, UK), and FTIR (Nicolet iS20, Thermo Fisher Scientific, USA), respectively. Oxidized konjac glucomannan was prepared by sodium periodate oxidation and verified by FTIR. The microstructure of freeze-dried hydrogels was observed by SEM, and Zn/Ca distribution was examined by EDS mapping. Hydrogel porosity was measured by mercury intrusion porosimetry. FTIR, XPS, and 2-dimensional correlation FTIR were used to analyze the chemical composition and molecular interactions within the hydrogel network.

Compression tests were performed on an Instron 5567 universal testing machine (Instron, USA) at a crosshead speed of 5 mm/min. Rheological properties were measured using a rheometer by recording the storage modulus, loss modulus, and viscosity. The wet adhesive properties of the hydrogels were evaluated using PBS-pretreated bovine bone specimens. The bone pieces were immersed in PBS for 1 h before testing. An equal amount of each hydrogel was placed between 2 bone pieces with a fixed bonding area and allowed to form in situ. The bonded specimens were subjected to tensile loading using a universal testing machine until interfacial failure occurred. Wet adhesive strength was calculated by dividing the maximum detachment force by the bonded area. Hydrogel specimens with identical dimensions were immersed in PBS for 1 h before mechanical testing. After removal of excess surface liquid, the hydrated samples were compressed using a universal testing machine at room temperature. The compressive modulus was calculated from the slope of the initial linear region of the stress–strain curve. All hydrogel groups were tested under the same loading conditions. Equal amounts of ZIF-8 were incubated in PBS at pH 7.4 or 6.5 at 37 °C. At predetermined time points, the remaining particles were collected, washed, freeze-dried, and weighed. The residual mass percentage was calculated relative to the initial dry weight. All measurements were performed in triplicate. Freeze-dried hydrogels were incubated in PBS at 37 °C, collected at predetermined time points, lyophilized, and weighed. The residual mass ratio was calculated as *W*_t_/*W*_0_ × 100%. For 6-gingerol release, GOZ6 hydrogels were incubated in PBS at 37 °C (pH 7.4 and 6.5), and the released 6-gingerol was quantified by high-performance liquid chromatography (HPLC). Samples containing CaO₂ were immersed in 10 ml of PBS. Dissolved oxygen was measured at predetermined time points using a dissolved oxygen meter (JB608, Remagnet, Shanghai, China). After each measurement, the release medium was replaced with fresh PBS. Blank PBS values were subtracted, and cumulative oxygen generation was calculated over 14 d.

### Cell culture and cytocompatibility evaluation

BMSCs were cultured in α-minimum essential medium (α-MEM, Thermo Fisher Scientific, Waltham, MA, USA) supplemented with 10% fetal bovine serum (FBS; Thermo Fisher Scientific) and 1% penicillin/streptomycin. RAW264.7 macrophages were cultured in Dulbecco’s modified Eagle’s medium (DMEM) containing 10% FBS and 1% penicillin/streptomycin. HUVECs were cultured in endothelial cell medium. All cells were maintained in a humidified incubator at 37 °C with 5% CO_2_. Before in vitro experiments, all hydrogel samples were sterilized by soaking in 75% ethanol followed by ultraviolet irradiation, and then thoroughly rinsed with sterile PBS. Hydrogel extracts were subsequently prepared for the following cell experiments. To evaluate cytocompatibility, BMSCs and RAW264.7 cells were separately seeded in culture plates and treated with hydrogel extracts from different hydrogel groups for 48 h. Cell viability was assessed using a Live/Dead staining kit (Beyotime, Haimen, China), and images were acquired using an inverted fluorescence microscope (Zeiss, Oberkochen, Germany). Cell proliferation was evaluated using a CCK-8 (Beyotime) assay. At the predetermined time points, the working solution was added to each well and incubated at 37 °C for 2 h, after which the absorbance at 450 nm was measured using a PowerWave XS spectrophotometer (BioTek, Winooski, VT, USA). The groupings for the cellular and animal experiments are shown in Table S1.

### Macrophage phenotype identification and metabolic analysis

To simulate the diabetic inflammatory microenvironment, RAW264.7 cells were cultured in high-glucose medium (35 mM glucose) supplemented with lipopolysaccharide (LPS; 1μg/ml). Cells were directly cocultured with extracts from different material groups and then collected for subsequent phenotype analysis. Immunofluorescence staining was performed to assess macrophage phenotypic changes. After different treatments, RAW264.7 cells were fixed with 4% paraformaldehyde (Sigma-Aldrich), permeabilized with 0.1% Triton X-100, and blocked with 5% bovine serum albumin (BSA). The cells were then incubated overnight with primary antibodies against CD86 and CD206. After washing, the corresponding fluorescent secondary antibodies were added and incubated for 1 h at room temperature in the dark, followed by nuclear counterstaining with 4′,6-diamidino-2-phenylindole (DAPI; Thermo Fisher Scientific). Images were captured using a fluorescence microscope and semiquantitatively analyzed using ImageJ.

Flow cytometry was used to quantify macrophage phenotypes. Cells from each group were collected and incubated with fluorescein isothiocyanate (FITC)-conjugated anti-mouse/human CD11b, Brilliant Violet 421-conjugated anti-mouse CD86, and phycoerythrin (PE)-conjugated anti-mouse CD206 antibodies for 30 min at 4 °C in the dark. After staining, the samples were analyzed by flow cytometry, and the data were processed using FlowJo software. A stepwise gating strategy was applied. First, the major cell population was selected on a forward scatter-area (FSC-A) versus side scatter-area (SSC-A) plot, and low-scatter events in the lower-left region were excluded as cellular debris. Singlets were then identified on an FSC-A versus FSC-H plot by retaining events distributed along the diagonal and excluding doublets and larger aggregates. Macrophage phenotypes were subsequently evaluated using CD11b versus CD86 and CD11b versus CD206 plots. Cells in the upper-right quadrant were defined as CD11b^+^CD86^+^ and CD11b^+^CD206^+^ macrophages, respectively. The same gating criteria were applied to all experimental groups.

In addition, RT-qPCR was performed to determine the relative mRNA expression levels of the pro-inflammatory genes Tnfα and Inos and the anti-inflammatory genes Tgfβ and Il10. ELISA kits were used to measure the secretion levels of TNF-α, IL-10, IL-12, and IFN-γ in the culture supernatants. To evaluate the effects of GOZ6 on macrophage metabolic status, glucose consumption, lactate production, and ATP levels in different treatment groups were measured using corresponding assay kits. Total cellular DNA was extracted, and the relative mitochondrial DNA (mtDNA) copy number was determined to comprehensively reflect cellular energy metabolism and mitochondrial function.

### RNA sequencing and mechanistic validation

To investigate the molecular mechanism by which GOZ6 regulates macrophages, RAW264.7 cells from the control group and the GOZ6-treated group were collected under diabetic inflammatory conditions. Total RNA was extracted using TRIzol reagent (Thermo Fisher Scientific), and cDNA libraries were constructed and sequenced on the Illumina NovaSeq platform. Differentially expressed genes were identified and further subjected to Gene Ontology (GO) and KEGG enrichment analyses, together with GSEA, to determine the altered biological processes and signaling pathways. Candidate signaling proteins were selected based on the transcriptomic and pathway analyses. Molecular docking was subsequently performed to predict possible binding conformations, relative binding energies, and potential interaction residues between 6-gingerol and the selected proteins. The docking analysis was considered hypothesis-generating and was not interpreted as evidence of direct binding or target engagement.

To further validate the key signaling axes, JAK1- and STAT3-deficient RAW264.7 macrophage models were established. Briefly, single-guide RNA sequences targeting the exons of the genes of interest were designed using the CRISPR-Cas9 system and cloned into the pSpCas9-2A-GFP (green fluorescent protein) vector, followed by transfection into RAW264.7 cells. Forty-eight hours after transfection, GFP-positive cells were sorted by flow cytometry. Gene editing efficiency was verified at the genomic level by PCR amplification and sequencing, and the knockout efficiency of JAK1 and STAT3 was further confirmed at the protein level by Western blotting. Thereafter, total protein was collected from wild-type and knockout RAW264.7 cells after different treatments, and Western blot analysis was performed to detect the expression of phenotype- and signaling-related proteins.

### In vitro angiogenesis-related evaluation

To evaluate the effects of direct material stimulation and macrophage-mediated paracrine regulation on endothelial recruitment and angiogenesis, both direct extract treatment experiments and macrophage-conditioned medium-mediated experiments were performed. In the direct treatment experiments, HUVECs were treated with extracts from different material groups and then subjected to scratch wound healing assays, Transwell migration assays, tube formation assays, and CD31 immunofluorescence staining. For the scratch assay, a standardized linear wound was created on confluent HUVECs monolayers using a sterile 200-μl pipette tip, and images were recorded at 0, 6, and 12 h to monitor cell migration. For the Transwell migration assay, HUVECs were seeded in the upper chamber, while extracts from different material groups were added to the lower chamber. After 24 h of incubation, cells that migrated to the lower surface of the membrane were fixed, stained with 0.5% crystal violet, and quantified using ImageJ. For the tube formation assay, HUVECs under the corresponding treatment conditions were seeded onto growth factor-reduced Matrigel, and capillary-like network formation was observed and quantified after 6 h. CD31 immunofluorescence staining was further performed to confirm the angiogenic phenotype of endothelial cells.

In the macrophage-conditioned medium-mediated experiments, RAW264.7 cells were first cocultured with extracts from different material groups for 24 h, and the supernatants were collected as conditioned media. These conditioned media were then used to treat HUVECs, followed by scratch, Transwell migration, tube formation, and CD31 immunofluorescence assays as described above, in order to evaluate the indirect regulatory effect of GOZ6-mediated macrophage reprogramming on endothelial angiogenic behavior.

### In vitro osteogenic evaluation and intracellular ROS and mitochondrial membrane potential assays

To evaluate the direct effects of the materials and the macrophage-mediated effects on the osteogenic differentiation of BMSCs, both direct treatment experiments and Transwell coculture experiments were performed. In the direct treatment experiments, BMSCs were cultured with extracts from different material groups for the assessment of intracellular ROS, mitochondrial membrane potential, and osteogenesis-related indices. To evaluate oxidative stress, BMSCs were cocultured with extracts from different material groups for 24 h and then stained using a DCFH-DA ROS detection kit (Beyotime, Haimen, China). Images were acquired using an inverted fluorescence microscope, and fluorescence intensity was semiquantitatively analyzed using ImageJ. Intracellular ROS levels were further quantified by flow cytometry. Flow cytometry data were analyzed using sequential gating. Cellular debris was excluded on an FSC-A versus SSC-A plot, and singlets were selected using FSC-A versus FSC-H. Intracellular ROS was then quantified according to FITC fluorescence and displayed as a histogram. An unstained control was used to define ROS-negative and ROS-positive populations. Identical gates were applied to all groups. Mitochondrial membrane potential was evaluated using a JC-1 fluorescent probe (Beyotime, Haimen, China). After treatment with different hydrogel extracts for 24 h, BMSCs were stained according to the manufacturer’s instructions, observed by fluorescence microscopy, and further quantified by flow cytometry. For JC-1 analysis, debris and aggregates were excluded using FSC-A versus SSC-A and FSC-A versus FSC-H gating. FITC and PE fluorescence were displayed on a 2-dimensional dot plot. Cells in the upper-right quadrant (FITC^+^PE^+^) were classified as having preserved mitochondrial membrane potential, whereas cells in the lower-right quadrant (FITC^+^PE^−^) were classified as depolarized. The same gating parameters were applied to all samples.

For the macrophage-mediated experiments, a Transwell coculture system was established. RAW264.7 cells were first treated with extracts from different hydrogels for 24 h and then seeded into the upper chamber of the Transwell system. BMSCs were seeded in the lower chamber and cultured in osteogenic induction medium to evaluate the effects of material-regulated macrophages on the osteogenic differentiation of BMSCs. Osteogenic differentiation was evaluated by ALP staining, ARS staining, and immunofluorescence staining. For ALP staining, cells were fixed and incubated with the ALP staining solution (Sigma-Aldrich) at room temperature in the dark for 30 min, followed by microscopic observation. For ARS staining, cells were fixed and incubated with ARS solution (Sigma-Aldrich) for 30 min, rinsed with PBS, and then observed for calcium nodule formation. In the long-term induction experiments, early osteogenic activity was assessed on day 7 using a bromochloroindolyl phosphate/nitro blue tetrazolium (BCIP/NBT) ALP staining kit, and ALP activity was quantified using an ALP assay kit (Sigma-Aldrich). On day 21, mineralization was evaluated by ARS staining, and the calcium nodules were dissolved in 10% cetylpyridinium chloride, followed by absorbance measurement at 560 nm.

For immunofluorescence staining of osteogenesis-related proteins, BMSCs were fixed with 4% paraformaldehyde for 15 min, blocked with Tris-buffered saline with Tween-20 (TBST) containing 5% BSA for 1 h, and incubated overnight at 4 °C with anti-collagen I antibody (1:500, ABclonal, Wuhan, China). After washing, goat anti-rabbit secondary antibody was added and incubated for 1 h at room temperature in the dark, followed by DAPI nuclear staining and fluorescence imaging. RT-qPCR was used to detect the expression levels of osteogenesis-related genes, including *Runx2, Col1a1, Alpl, Spp1*, and *Bglap*.

### Quantitative real-time PCR

Total RNA from cells or tissues was extracted using the FastPure Cell/Tissue Total RNA Isolation Kit (Vazyme, R401), and RNA concentration was measured using a NanoDrop spectrophotometer. One microgram of RNA was reverse-transcribed using HiScript III RT SuperMix for qPCR (Vazyme, R323). Quantitative real-time PCR was performed using ChamQ Universal SYBR qPCR Master Mix (Vazyme, Q411) on a QuantStudio 6 Flex real-time PCR system (Applied Biosystems). *C*_t_ values were collected using the supporting software and analyzed by the 2^−ΔΔ*C*t^ method, with β-actin used as the internal reference gene. The qPCR primer sequences are provided in Tables S2 and S3.

### Establishment and evaluation of the diabetic rat calvarial defect model

All animal experiments were conducted in accordance with institutional ethical guidelines and were approved by the animal ethics committee (Ethics approval no. SUDA20251107A05). Six-week-old male Sprague–Dawley rats were fed a high-fat diet for 4 weeks. Streptozotocin (STZ) was administered at a dose of 30 mg/kg via intraperitoneal injection for 3 consecutive days. Rats with fasting blood glucose levels continuously higher than 16.7 mM were considered successfully modeled. Under general anesthesia, bilateral full-thickness critical-sized calvarial defects (5 mm in diameter) were created using a sterile trephine bur, and different materials were implanted into the defect sites according to the experimental grouping. Animals were divided into 5 groups: Blank control, GO, GOZ, GO6, and GOZ6. In the Blank control group, calvarial defects were created without implantation of any material. Animals were sacrificed at 4 and 8 weeks postoperatively, and specimens were harvested for subsequent imaging and histological analyses.

Harvested calvarial specimens were scanned using high-resolution micro-CT to evaluate the 3-dimensional morphology of newly formed bone. BMD and BV/TV within the defect area were quantitatively analyzed. After routine decalcification, embedding, and sectioning, specimens were subjected to hematoxylin and eosin (H&E) staining and Masson’s trichrome staining to evaluate tissue repair and collagen deposition within the defect region. To further assess the in vivo immunoregulatory, osteogenic, and angiogenic effects, immunohistochemical staining was performed to detect the expression of TNF-α, IL-10, COL1A1, CD31, and RUNX2 in the defect area. At 8 weeks after surgery, major organs including the heart, liver, spleen, lung, and kidney were harvested, paraffin-embedded, sectioned, and stained with H&E to evaluate the systemic biosafety of the implanted materials.

### Statistical analysis

Data are presented as the mean ± SD. Statistical analyses were performed using GraphPad Prism 10.1.2. Comparisons among independent groups were conducted using ordinary one-way analysis of variance (ANOVA) followed by Tukey’s multiple-comparisons test for all pairwise comparisons. A value of *P* < 0.05 was considered statistically significant.

## Ethical Approval

All animal experiments were conducted in accordance with institutional guidelines and were approved by the institutional Animal Ethics Committee (approval no. SUDA20251107A05).

## Data Availability

Data can be obtained by contacting the corresponding author.
