# Supplementary material for: A pH-Responsive Polysaccharide Hydrogel Modulates Oxygen Delivery and Immunometabolic Remodeling for Diabetic Bone Regeneration
Source: Research (Wash D C). 2026 Oct 5;9:1428. doi: 10.34133/research.1428 (PMC13636438; doi:10.34133/research.1428)
Supplement: Supplementary file 1 [file research.1428.f1.docx]

**Supplementary Fig. 1.** SEM photographs and EDS elemental mapping of CaO_2_.

**Supplementary Fig. 2.** EDS elemental mapping of ZIF-8.

**Supplementary Fig. 3.** EDS elemental mapping of ZIF-8@CaO_2_.

**Supplementary Fig. 4.** Particle Size Analysis of CaO_2_.

**Supplementary Fig. 5.** TEM photographs and EDS elemental mapping of CaO_2_.

**Supplementary Fig. 6.** EDS elemental mapping of ZIF-8.

**Supplementary Fig. 7.** XPS spectra acquired for the ZIF-8

**Supplementary Fig. 8. Remaining mass of ZIF-8 particles after incubation in PBS at pH 7.4 or 6.5.** Data are presented as the mean ± SD (n = 3).

**Supplementary Fig. 9.** GOZ6 Hydrogel Adhesion Diagram.

**Supplementary Fig. 10.** EDS elemental mapping of GO, GO6, GOZ.

**Supplementary Fig. 11.** **Oxygen-generation profiles of free CaO₂, GO@CaO₂, and GOZ6 in PBS.** Data are presented as the mean ± SD (n = 3).

**Supplementary Fig. 12. a) Young’s Modulous of the hydrogels. b) Adhesive Strength of the hydrogels.** Data are presented as the mean ± SD (n = 3). Compared with the GO group: **p* < 0.05, ***p* < 0.01, ****p* < 0.001, ns: not significant (*p* > 0.05).

**Supplementary Fig. 13.** Cytocompatibilities of the spun GO, GOZ, GO6 and GOZ6. **a)** Living cells (green) and dead cells (red) of BMSCs co-cultured with the hydrogel’ system. Scale bars: 400μm. **b)** Semi-quantification of cell viability of BMSCs based on Live/Dead staining. **c)** Cell viability of BMSCs evaluated by CCK-8 assay. Data are presented as the mean ± SD (n = 5).

**Supplementary Fig. 14.** Cytocompatibilities of the spun GO, GOZ, GO6 and GOZ6. **a)** Living cells (green) and dead cells (red) of RAW264.7 cells co-cultured with the hydrogel’ system. Scale bars: 400μm. **b)** Semi-quantification of cell viability of RAW264.7 cells based on Live/Dead staining. **c)** Cell viability of RAW264.7 cells evaluated by CCK-8 assay. Data are presented as the mean ± SD (n = 5).

**Supplementary Fig. 15.** **a-b)** Flow cytometric analysis of the proportion of M1 and M2 macrophage. Data are presented as the mean ± SD (n = 5). Compared with the GO group: **p* < 0.05, ***p* < 0.01, ****p* < 0.001, ns: not significant (*p* > 0.05). Compared with the GOZ6 group: ^#^*p* < 0.05, ^##^*p* < 0.01, ^###^*p* < 0.001, ns: not significant (*p* > 0.05).

**Supplementary Fig. 16.** **ELISA detection of IL-12 expression levels.** Data are presented as the mean ± SD (n = 5). Compared with the GO group: **p* < 0.05, ***p* < 0.01, ****p* < 0.001, ns: not significant (*p* > 0.05).

**Supplementary Fig. 17.** RT-qPCR analysis of *Inos* expression in RAW264.7 cells under different treatments. Data are presented as the mean ± SD (n = 5). Compared with the GO group: **p* < 0.05, ***p* < 0.01, ****p* < 0.001, ns: not significant (*p* > 0.05). Compared with the GOZ6 group: ^#^*p* < 0.05, ^##^*p* < 0.01, ^###^*p* < 0.001, ns: not significant (*p* > 0.05).

**Supplementary Fig. 18.** Representative immunoblotting results showing regulation of the IL-10 in RAW264.7 cells under different treatment conditions.

**Supplementary Fig. 19.** Fluorescence intensity of JC-1. Data are presented as the mean ± SD (n = 5). Compared with the GO group: **p* < 0.05, ***p* < 0.01, ****p* < 0.001, ns: not significant (*p* > 0.05). Compared with the GOZ6 group: ^#^*p* < 0.05, ^##^*p* < 0.01, ^###^*p* < 0.001, ns: not significant (*p* > 0.05).

**Supplementary Fig. 20.** Percentages of FITC in BMSCs cultured on the different material groups, as determined by flow cytometry. Data are presented as the mean ± SD (n = 5). Compared with the GO group: **p* < 0.05, ***p* < 0.01, ****p* < 0.001, ns: not significant (*p* > 0.05). Compared with the GOZ6 group: ^#^*p* < 0.05, ^##^*p* < 0.01, ^###^*p* < 0.001, ns: not significant (*p* > 0.05).

**Supplementary Fig. 21.** Fluorescence intensity of ROS. Data are presented as the mean ± SD (n = 5). Compared with the GO group: **p* < 0.05, ***p* < 0.01, ****p* < 0.001, ns: not significant (*p* > 0.05). Compared with the GOZ6 group: ^#^*p* < 0.05, ^##^*p* < 0.01, ^###^*p* < 0.001, ns: not significant (*p* > 0.05).

**Supplementary Fig. 22.** **a)** Flow cytometric analysis of intracellular ROS levels in BMSCs. **b)** Percentages of Fluo-FITC-A+ BMSCs cultured on the different material groups, as determined by flow cytometry. Data are presented as the mean ± SD (n = 5). Compared with the GO group: **p* < 0.05, ***p* < 0.01, ****p* < 0.001, ns: not significant (*p* > 0.05). Compared with the GOZ6 group: ^#^*p* < 0.05, ^##^*p* < 0.01, ^###^*p* < 0.001, ns: not significant (*p* > 0.05).

**Supplementary Fig. 23.** RT-qPCR analysis of *Bglap* expression in BMSCs under different treatments. Data are presented as the mean ± SD (n = 5). Compared with the GO group: **p* < 0.05, ***p* < 0.01, ****p* < 0.001, ns: not significant (*p* > 0.05). Compared with the GOZ6 group: ^#^*p* < 0.05, ^##^*p* < 0.01, ^###^*p* < 0.001, ns: not significant (*p* > 0.05).

**Supplementary Fig. 24.** H&E staining of lung, kidney, spleen, liver and heart at 8 weeks after the implantation of scaffolds. Scale bars=100μm.

**Supplementary Table 1.** Material grouping.

| Group | Denoted | Function |
| --- | --- | --- |
| Gelatin and OKGM are cross-linked via Schiff base to form a GO hydrogel | GO | Control |
| ZIF-8@CaO₂ was uniformly incorporated into the GO hydrogel | GOZ | Control |
| 6-Gingerol was uniformly incorporated into the GO hydrogel | GO6 | Control |
| 6-Gingerol and ZIF-8@CaO₂ were uniformly incorporated into the GO hydrogel | GOZ6 | Test |

**Supplementary Table 2.** Primer sequences for mouse RAW cells.

| Gene | Forward primer sequence (5ʹ -3ʹ) | Reverse primer sequence (5ʹ -3ʹ) |
| --- | --- | --- |
| *β-actin* | GGCTGTATTCCCCTCCATCG | CCAGTTGGTAACAATGCCATGT |
| *TGF-β* | TGGAGCAACATGTGGAACTC | TGCCGTACAACTCCAGTGAC |
| *IL-10* | GGTGAGAAGCTGAAGACCCT | CACTCTTCACCTGCTCCACTG |
| *TNF-α* | CAACGGCATGGATCTCAAAG | CAGCCTTGTCCCTTGAAGA |
| *iNOS* | ATGCTACTGGAGGTGGGTG | GATGTTGTAGCGCTGTGTGT |

**Supplementary Table 3.** Primer sequences for rat BMSCs.

| Gene | Forward primer sequence (5ʹ -3ʹ) | Reverse primer sequence (5ʹ -3ʹ) |
| --- | --- | --- |
| *β-actin* | CCCGCGAGTACAACCTTCT | CGTCATCCATGGCGAACT |
| *Alpl* | TATGTCTGGAACCGCACTGAAC | CACTAGCAAGAAGAAGCCTTTGG |
| *Col1a1* | CAGGCTGGTGTGATGGGATT | CCAAGGTCTCCAGGAACACC |
| *Bglap* | AACGGTGGTGCCATAGATGC | AGGACCCTCTCTCTGCTCAC |
| *Runx2* | ATCCAGCCACCTTCACTTACACC | GGGACCATTGGGAACTGATAGG |
